# Supplementary material for: Improving risk analysis of the environmental drivers of the spillover, emergence/re-emergence and spread of Crimean-Congo haemorrhagic fever virus, Marburg virus and Middle East respiratory syndrome coronavirus in the East Africa Region
Source: BMJ Glob Health. 2025 Apr 16;10(4):e019162. doi: 10.1136/bmjgh-2025-019162 (PMC12004484; doi:10.1136/bmjgh-2025-019162)
Supplement: online supplemental file 1 [file bmjgh-10-4-s001.docx]

**SUPPLEMENTAL MATERIALS**

Supplemental Table 1 (ST1): Summary of reported outbreaks of Crimean-Congo haemorrhagic fever (CCHF) and Marburg virus disease (MVD) and evidence of Crimean-Congo haemorrhagic fever virus (CCHFV) and Middle East respiratory syndrome coronavirus (MERS-CoV) seroprevalence across countries in the East Africa region

| Pathogen | Disease | Year of Outbreak | Country of origin | Source of Transmission |
| --- | --- | --- | --- | --- |
| CCHFV | CCHF | 2022^1^ | Uganda | Human |
|  |  | 2018-2019^2^ | Uganda | Human |
|  |  | 2018^3^ | Uganda | Human |
|  |  | 2013 - 2017^3^ | Uganda | Human |
|  |  | 2015 - 2016^4,5^ | Sudan | Nosocomial and Human |
|  |  | 2008 - 2009^6^ | Madagascar | Tick and Human |
|  |  | 2009^7^ | Sudan | Nosocomial and Human |
|  |  | 2008^7^ | Sudan | Nosocomial and Human |
|  |  | 2000^8^ | Kenya | Tick and Human |
| MARV | MVD | 2024^9^ | Rwanda | Cave visit |
|  |  | 2023^10^ | Tanzania | Inconclusive |
|  |  | 2007^11^ | Uganda | Kitaka Mines |
|  |  | 2017^12^ | Uganda | Mines and cave visits |
|  |  | 2014^13^ | Uganda | Mines and cave visits |
|  |  | 2012^14,15^ | Uganda | Mine and cave visits |
|  |  | 2008 (July)^16^ | Uganda | Gold mine and cave visits |
|  |  | 2008 (January)^17^ | Uganda | Cave visits |
|  | | | | |
| Pathogen | **Disease** | **Year** | **Location** | **Evidence of Seroprevalence** |
| CCHFV | CCHFV | 2024^18^ | Northern Tanzania | 15.1-49.6% |
|  | CCHFV | 2021^19^ | Kenya |  |
|  | CCHFV | January 2020 – December 2021^20^ | Kenya | 11.9 % seroprevalence among livestock [donkeys [31.4%), cattle (14.1%), sheep (9.8%), goats (8.1%)] |
|  | CCHFV | 2012^21^ | Kenya |  |
|  | MERS | 2018 – 2020^22,23^ | Kenya | 0.2 % seroprevalence (3 humans tested positive); 100% for camels |
| MERS-CoV |  | January 2016 – June 2018^24^ | Across Kenya | 82% seroprevalence among adult camels |
|  |  | January 2016 – June 2018^22,24^ | All (13) camel-rearing counties in Kenya | 68% seroprevalence across all counties combined |
|  |  | 2016-2018^25^ | Northeast Region, Uganda | 66% and 65% seroprevalence in camels from Morto and Amudat respectively |
|  |  | 2017^26^ | Somalia | 100% seroprevalence in camel sera |
|  |  | 2017^26^ | Sudan | 100% seroprevalence in camels |
|  |  | October – November 2016^27^ | Kenya | 8.6% seroprevalence among camel herders |
|  |  | 2016^28^ | Ethiopia | 70% seroprevalence in camels |
|  |  | 2015^26^ | Sudan | 92% seroprevalence in camels |
|  |  | 2013 - 2014^29^ | Garissa and Tana River counties, Kenya | Evidence of seropositivity in humans |
|  |  | 2013^30,31^ | Marsabit county, Kenya | 90% seroprevalence among camels |
|  |  | 2010 - 2011^32^ | Ethiopia | 93% and 97% seropositivity reported in Juvenile and adult camels respectively |

*Supplemental Table 2 (ST2): List of review search terms and inclusion criteria by questions*

| **Review Question** | **Database Search terms** |
| --- | --- |
| 1. **What are the environmental drivers of the spillover, emergence/reemergence, and spread of Crimean-Congo haemorrhagic fefer virus (CCHFV), Marburg virus (MARV), and Middle East respiratory syndrome coronavirus (MERS-CoV) in the East Africa region?**   Inclusion criteria   - Environmental drivers - Outcome of Interest (CCHFV/Crimean-Congo haemorrhagic fever (CCHF), MARV/Marburg virus disease (MVD), MERS-CoV/Middle East respiratory syndrome (MERS) - East Africa - English Language | **Pubmed**  "Africa, Eastern"[Mesh] OR "Africa east"[tiab:~2] OR "Africa eastern"[tiab:~2] OR "African east"[tiab:~2] OR "African eastern"[tiab:~2] OR "africans east"[tiab:~2] OR "africans eastern"[tiab:~2] OR Burundi*[tw] OR Comoros*[tw] OR Djibouti*[tw] OR Eritrea*[tw] OR Ethiopia*[tw] OR Kenya*[tw] OR Madagascar*[tw] OR Mauritius* [tw] OR Rwanda*[tw] OR Seychelles*[tw] OR Somalia*[tw] OR Sudan*[tw] OR Tanzania*[tw] OR Uganda*[tw] OR "Addis Ababa"[tw] OR Antananarivo[tw] OR Asmara[tw] OR Bujumbura[tw] OR "Dar es Salaam"[tw] OR "Dire Dawa"[tw] OR "Djibouti City"[tw] OR Dodoma[tw] OR Gitega[tw] OR Hargeisa[tw] OR Juba[tw] OR Kampala[tw] OR Keren[tw] OR Khartoum[tw] OR Kigali[tw] OR Kisumu[tw] OR Lindi[tw] OR Mahajanga[tw] OR Mbarara[tw] OR Mogadishu[tw] OR Mombasa[tw] OR Muhanga[tw] OR Moroni[tw] OR Muyinga[tw] OR Mwanza[tw] OR Nairobi[tw] OR Ngozi[tw] OR Omdurman[tw] OR "Port Louis"[tw] OR Victoria[tw] OR "Great Lakes Region*"[tw] OR "Horn of Africa*"[tw] OR "Nile Valle*"[tw]  AND  "Hemorrhagic Fever Virus, Crimean-Congo"[Mesh] OR "Crimean-congo hemorrhagic fever*"[tw] OR "Crimean-Congo haemorrhagic fever*"[tw] OR CCHF[tw] OR "congo virus*"[tw] OR "Crimean hemorrhagic fever*"[tw] OR "Marburg Virus Disease"[Mesh] OR Marburg[tw] OR MVD[tw] OR "Middle East Respiratory Syndrome Coronavirus"[Mesh] OR "Middle East Respiratory syndrome coronavirus"[tw] OR "MERS-CoV"[tw] OR "MERS Virus*"[tw] OR "Middle East respiratory syndrome"[tw] OR Merbecovirus*[tw]  **Embase**  ‘east african’/exp OR ((Africa* NEAR/2 east*) OR Burundi* OR Comoros* OR Djibouti* OR Eritrea* OR Ethiopia* OR Kenya* OR Madagascar* OR Mauritius* OR Rwanda* OR Seychelles* OR Somalia* OR Sudan* OR Tanzania* OR Uganda* OR “Addis Ababa” OR Antananarivo OR Asmara OR Bujumbura OR “Dar es Salaam” OR “Dire Dawa” OR “Djibouti City” OR Dodoma OR Gitega OR Hargeisa OR Juba OR Kampala OR Keren OR Khartoum OR Kigali OR Kisumu OR Lindi OR Mahajanga OR Mbarara OR Mogadishu OR Mombasa OR Muhanga OR Moroni OR Muyinga OR Mwanza OR Nairobi OR Ngozi OR Omdurman OR “Port Louis” OR Victoria OR “Great Lakes Region*” OR “Horn of Africa*” OR “Nile Valle*”):ab,ti,kw  AND  'Crimean-Congo hemorrhagic fever virus'/exp OR 'Marburg hemorrhagic fever'/exp OR 'Middle East respiratory syndrome coronavirus'/exp OR ("Crimean-congo hemorrhagic fever*" OR "Crimean-Congo haemorrhagic fever*" OR CCHF OR "congo virus*" OR "Crimean hemorrhagic fever*" OR Marburg OR MVD OR "Middle East Respiratory syndrome coronavirus" OR "MERS-CoV" OR "MERS Virus*" OR "Middle East respiratory syndrome" OR Merbecovirus*):ab,ti,kw  **Scopus**  TITLE-ABS-KEY ((Africa* W/2 east*) OR Burundi* OR Comoros* OR Djibouti* OR Eritrea* OR Ethiopia* OR Kenya* OR Madagascar* OR Mauritius* OR Rwanda* OR Seychelles* OR Somalia* OR Sudan* OR Tanzania* OR Uganda* OR "Addis Ababa" OR Antananarivo OR Asmara OR Bujumbura OR "Dar es Salaam" OR "Dire Dawa" OR "Djibouti City" OR Dodoma OR Gitega OR Hargeisa OR Juba OR Kampala OR Keren OR Khartoum OR Kigali OR Kisumu OR Lindi OR Mahajanga OR Mbarara OR Mogadishu OR Mombasa OR Muhanga OR Moroni OR Muyinga OR Mwanza OR Nairobi OR Ngozi OR Omdurman OR "Port Louis" OR Victoria OR "Great Lakes Region*" OR "Horn of Africa*" OR "Nile Valle*")  AND  TITLE-ABS-KEY ("Crimean-congo hemorrhagic fever*" OR CCHF OR "congo virus*" OR "Crimean hemorrhagic fever*" OR "Crimean-Congo haemorrhagic fever*" OR Marburg OR MVD OR "Middle East Respiratory syndrome coronavirus" OR "MERS-CoV" OR "MERS Virus*" OR "Middle East respiratory syndrome" OR Merbecovirus*)  **Web of Science**  TS=("Crimean-congo hemorrhagic fever*" OR CCHF OR "congo virus*" OR "Crimean hemorrhagic fever*" OR "Crimean-Congo haemorrhagic fever*" OR Marburg OR MVD OR "Middle East Respiratory syndrome coronavirus" OR "MERS-CoV" OR "MERS Virus*" OR "Middle East respiratory syndrome" OR Merbecovirus*)  AND  TS=((Africa* NEAR/2 east*) OR Burundi* OR Comoros* OR Djibouti* OR Eritrea* OR Ethiopia* OR Kenya* OR Madagascar* OR Mauritius* OR Rwanda* OR Seychelles* OR Somalia* OR Sudan* OR Tanzania* OR Uganda* OR "Addis Ababa" OR Antananarivo OR Asmara OR Bujumbura OR "Dar es Salaam" OR "Dire Dawa" OR "Djibouti City" OR Dodoma OR Gitega OR Hargeisa OR Juba OR Kampala OR Keren OR Khartoum OR Kigali OR Kisumu OR Lindi OR Mahajanga OR Mbarara OR Mogadishu OR Mombasa OR Muhanga OR Moroni OR Muyinga OR Mwanza OR Nairobi OR Ngozi OR Omdurman OR "Port Louis" OR Victoria OR "Great Lakes Region*" OR "Horn of Africa*" OR "Nile Valle*")  **CAB Direct and CABI Digital Library**  ("Crimean-congo hemorrhagic fever" OR "Crimean-congo hemorrhagic fevers" OR "Crimean-Congo haemorrhagic fever" OR "Crimean-Congo haemorrhagic fevers" OR CCHF OR "congo virus" OR "congo viruses" OR "Crimean hemorrhagic fever" OR "Crimean hemorrhagic fevers" OR Marburg OR MVD OR "Middle East Respiratory syndrome coronavirus" OR "MERS-CoV" OR "MERS Virus" OR "MERS Viruses" OR "Middle East respiratory syndrome" OR Merbecovirus*)  AND  ("East Africa" OR "Eastern Africa" OR "East African" OR "Eastern African" OR Burundi* OR Comoros* OR Djibouti* OR Eritrea* OR Ethiopia* OR Kenya* OR Madagascar* OR Mauritius* OR Rwanda* OR Seychelles* OR Somalia* OR Sudan* OR Tanzania* OR Uganda* OR "Addis Ababa" OR Antananarivo OR Asmara OR Bujumbura OR "Dar es Salaam" OR "Dire Dawa" OR "Djibouti City" OR Dodoma OR Gitega OR Hargeisa OR Juba OR Kampala OR Keren OR Khartoum OR Kigali OR Kisumu OR Lindi OR Mahajanga OR Mbarara OR Mogadishu OR Mombasa OR Muhanga OR Moroni OR Muyinga OR Mwanza OR Nairobi OR Ngozi OR Omdurman OR "Port Louis" OR Victoria OR "Great Lakes Region" OR "Great Lakes Regions" OR "Horn of Africa" OR "Nile Valle") |
| 1. **What risk frameworks and/or methodological tools exist for assessing and managing environmentally driven zoonotic emerging and/or reemerging infectious diseases (EIDs) and what are the gaps in existing biological threat risk analysis frameworks?**   Inclusion criteria   - Risk Analysis related - Biological threats - English language | **Pubmed**  **("Zoonoses"[Mesh] OR "Disease Outbreaks"[Mesh:NoExp] OR "Disease Hotspot"[Mesh] OR "Epidemics"[Mesh:NoExp] OR "Space-Time Clustering"[Mesh] OR "Disease Transmission, Infectious"[Mesh] OR "Animals"[Mesh] OR "Livestock"[Mesh] OR "Communicable Diseases, Emerging"[Mesh] OR "Communicable Diseases"[Mesh]** OR Zoono*[tw] OR "animal to human"[tiab:~5] OR "animal to humans"[tiab:~5] OR "animals to human"[tiab:~5] OR "animals to humans"[tiab:~5] OR "infectious disease outbreak"[tiab:~5] OR "infectious disease outbreaks"[tiab:~5] OR "infectious diseases outbreak"[tiab:~5] OR "infectious diseases outbreaks"[tiab:~5] OR epidemic*[tw] OR "disease transmission"[tw] OR "disease spread"[tw] OR "disease cluster*"[tw] OR "cluster of cases"[tw] OR "emerging diseases"[tiab:~5] OR "reemerging diseases"[tiab:~5]  OR "re emerging diseases"[tiab:~5] OR spillover*[tw] OR animal*[tw] OR livestock*[tw] OR (viral[tw] AND ("biological threat*"[tw] OR "bio-threat*"[tw])) OR "Hemorrhagic Fever Virus, Crimean-Congo"[Mesh] OR "Crimean-congo hemorrhagic fever*"[tw] OR "Crimean-Congo haemorrhagic fever*"[tw] OR CCHF[tw] OR "congo virus*"[tw] OR "Crimean hemorrhagic fever*"[tw] OR "Marburg Virus Disease"[Mesh] OR Marburg[tw] OR MVD[tw] OR "Middle East Respiratory Syndrome Coronavirus"[Mesh] OR "Middle East Respiratory syndrome coronavirus"[tw] OR "MERS-CoV"[tw] OR "MERS Virus*"[tw] OR "Middle East respiratory syndrome"[tw] OR Merbecovirus*[tw] )  AND  **("Risk Assessment"[Mesh] OR "Decision Making"[Mesh]** OR "risk assessment*"[tw] OR "risk analys*"[tw] OR "risk manag*"[tw] OR "risk communication*"[tw] OR "joint assessment"[tw] OR "risk mitigation"[tw] OR "mitigating risk*"[tw] OR "decision making"[tw] OR "assessment framework*"[tw] OR "analysis framework*"[tw] OR mitigation[tw] OR "integrated risk*"[tw] OR "risks hazard"[tiab:~3] OR "risks hazards"[tiab:~3] OR "risks hazardous"[tiab:~3])  AND  **("One Health"[Mesh]** OR "one health*"[tw] OR tripartite*[tw] OR "transdisciplinary health*"[tw] OR "trans disciplinary health*"[tw] OR "integrated health*"[tw] OR  (("human animal"[tiab:~3] OR "humans animals"[tiab:~3] OR "humans animal"[tiab:~3] OR "human animals"[tiab:~3]) AND (environment*[tw] OR ecosystem*[tw] OR "planetary health"[tw])) OR (("Human agriculture"[tiab:~3] OR "Humans agriculture"[tiab:~3] OR "Human agricultural"[tiab:~3] OR "Humans agricultural"[tiab:~3]  OR "Human livestock"[tiab:~3] OR "Humans livestock"[tiab:~3]) AND (environment*[tw] OR ecosystem*[tw] OR "planetary health"[tw])))  AND  **("Climatic Processes"[Mesh] OR "Ecological and Environmental Phenomena"[Mesh:NoExp] OR "Anthropogenic Effects"[Mesh] OR "Carbon Footprint"[Mesh] OR "Ecosystem"[Mesh] OR "Environment"[Mesh] OR "Environmental Health"[Mesh]** **OR "Meteorological Concepts"[Mesh] OR "Conservation of Natural Resources"[Mesh]** OR **"Disasters"[Mesh]** OR **"Climate Change"[Mesh]** **OR "Greenhouse Gases"[Mesh] OR "Ozone Depletion"[Mesh] OR "Carbon Footprint"[Mesh]**OR disaster*[tw] OR postdisaster*[tw] OR posthazard*[tw] OR hazard*[tw] OR avalanche*[tw] OR cyclonic storm*[tw] OR cyclone*[tw] OR hurricane*[tw] OR tropical storm*[tw] OR typhoon*[tw] OR drought*[tw] OR earthquake*[tw] OR flood*[tw] OR landslide*[tw] OR rockslide*[tw] OR mudslide*[tw] OR earth tide*[tw] OR ocean tide*[tw] OR tidalwave*[tw] OR tidal wave*[tw] OR tsunami*[tw] OR tornado*[tw] OR wildfire*[tw] OR wildland fire*[tw] OR brush fire*[tw] OR forest fire*[tw] OR wild fire*[tw] OR facility fire*[tw] OR volcanic*[tw] OR major rainfall*[tw] OR storm[tw] OR storms[tw] OR fire*[tw]  OR volcano*[tw] OR volcanic[tw] OR "extreme temperature*"[tw] OR "extreme weather*"[tw] OR heatwave*[tw] OR "heat wave*"[tw] OR "extreme heat*"[tw] OR "extreme cold*"[tw] OR rainfall[tw] OR humidity[tw] OR altitude[tw] OR climate*[tw] OR climatic[tw] OR "global warm*"[tw] OR "greenhouse effect*"[tw] OR "greenhouse gas"[tw] OR "green house effect*"[tw] OR "green house gas"[tw] OR "green house gases"[tw] OR "greenhouse gases"[tw] OR "ozone depletion"[tw] OR "ozone hole*"[tw] OR "carbon foot*"[tw]  OR deforestation[tw] OR reforestation[tw] OR logging[tw] OR conservation[tw] OR "carrying capacit*"[tw] OR "environmental protection"[tw] OR "environmental biodegradation"[tw] OR "sustainable development*"[tw] OR "smart growth"[tw] OR Meteorological[tw] OR Anthropogenic[tw] OR "human impact*"[tw] OR "land degradation"[tw] OR "forest clearing"[tw] OR "forest restoration"[tw] OR "carbon sink*"[tw] OR "ecosystem respiration"[tw] OR "carbon flux*"[tw] OR environment*[tw] OR livestock*[tw] OR temperature*[tw] OR warm*[tw] OR meteo*[tw] OR ecology[tw] OR ecologically[tw] OR ecological[tw] OR ecosystem**[tw] OR biodiversity[tw] OR "land use"[tw] OR wilderness[tw] OR wildlife[tw] OR forest*[tw])  **Embase**  **('zoonosis'/exp OR 'epidemic'/de OR 'disease hotspot'/exp OR 'spatiotemporal analysis'/exp OR 'zoonotic transmission'/exp OR 'pathogen transmission'/exp OR 'nosocomial transmission'/exp OR 'community transmission'/exp OR 'animal'/exp OR 'livestock'/exp OR 'communicable disease'/exp OR 'emerging infectious disease'/exp OR 'Marburg hemorrhagic fever'/exp OR 'Crimean-Congo hemorrhagic fever virus'/exp OR 'Middle East respiratory syndrome coronavirus'/exp) OR** (Zoono* OR epidemic* OR "disease transmission" OR "disease spread" OR "disease cluster*" OR "cluster of cases" OR (animal* NEAR/5 human*) OR ("infectious disease*" NEAR/5 outbreak*) OR (emerging NEAR/5 disease*) OR (reemerging NEAR/5 disease*) OR spillover* OR animal* OR livestock* OR (viral AND ("biological threat*" OR "bio-threat*")) OR "Crimean-congo hemorrhagic fever*" OR "Crimean-Congo haemorrhagic fever*" OR CCHF OR "congo virus*" OR "Crimean hemorrhagic fever*" OR Marburg OR MVD OR "Middle East Respiratory syndrome coronavirus" OR "MERS-CoV" OR "MERS Virus*" OR "Middle East respiratory syndrome" OR Merbecovirus*):ab,ti,kw  AND  **('risk assessment'/exp OR 'decision making'/exp)** OR ("risk assessment*" OR "risk analys*" OR "risk manag*" OR "risk communication*" OR "joint assessment" OR "risk mitigation" OR "mitigating risk*" OR "decision making" OR "assessment framework*" OR "analysis framework*" OR mitigation OR "integrated risk*" OR (risks NEAR/3 hazard*)):ab,ti,kw  AND  **('One Health'/exp)** OR ('one health*':ab,ti,kw OR tripartite*:ab,ti,kw OR 'transdisciplinary health*':ab,ti,kw OR 'trans disciplinary health*':ab,ti,kw OR 'integrated health*':ab,ti,kw) OR (((human* NEAR/3 (animal* OR agricultur* OR livestock*)):ab,ti,kw) AND (environment*:ab,ti,kw OR ecosystem*:ab,ti,kw OR 'planetary health':ab,ti,kw))  AND  **('climate change'/exp OR 'environmental aspects and related phenomena'/de OR 'environmental change'/exp OR 'environmental impact'/exp OR 'human impact (environment)'/exp OR 'carbon footprint'/exp OR 'ecosystem'/exp OR 'environment'/exp OR 'environmental health'/exp OR 'meteorological phenomena'/exp OR 'environmental protection'/exp OR 'disaster'/exp OR 'greenhouse gas emission'/exp OR 'ozone depletion'/exp OR 'carbon footprint'/exp)** OR (disaster* OR postdisaster* OR posthazard* OR hazard* OR avalanche* OR "cyclonic storm*" OR cyclone* OR hurricane* OR "tropical storm*" OR typhoon* OR drought* OR earthquake* OR flood* OR landslide* OR rockslide* OR mudslide* OR "earth tide*" OR "ocean tide*" OR tidalwave* OR "tidal wave*" OR tsunami* OR tornado* OR wildfire* OR "wildland fire*" OR "brush fire*" OR "forest fire*" OR "wild fire*" OR "facility fire*" OR volcanic* OR "major rainfall*" OR storm OR storms OR fire* OR volcano* OR volcanic OR "extreme temperature*" OR "extreme weather*" OR heatwave* OR "heat wave*" OR "extreme heat*" OR "extreme cold*" OR rainfall OR humidity OR altitude OR climate* OR climatic OR "global warm*" OR "greenhouse effect*" OR "greenhouse gas" OR "green house effect*" OR "green house gas" OR "green house gases" OR "greenhouse gases" OR "ozone depletion" OR "ozone hole*" OR "carbon foot*"  OR deforestation OR reforestation OR logging OR conservation OR "carrying capacit*" OR "environmental protection" OR "environmental biodegradation" OR "sustainable development*" OR "smart growth" OR Meteorological OR Anthropogenic OR "human impact*" OR "land degradation" OR "forest clearing" OR "forest restoration" OR "carbon sink*" OR "ecosystem respiration" OR "carbon flux*" OR environment* OR livestock* OR temperature* OR warm* OR meteo* OR ecology OR ecologically OR ecological OR ecosystem* OR biodiversity OR "land use" OR wilderness OR wildlife OR forest*):ab,ti,kw  AND  [2000-2023]/py  **Scopus**  TITLE-ABS-KEY((Zoono* OR epidemic* OR "disease transmission" OR "disease spread" OR "disease cluster*" OR "cluster of cases" OR (animal* W/5 human*) OR ("infectious disease*" W/5 outbreak*) OR (emerging W/5 disease*) OR (reemerging W/5 disease*) OR spillover* OR animal* OR livestock* OR (viral AND ("biological threat*" OR "bio-threat*")) OR "Crimean-congo hemorrhagic fever*" OR "Crimean-Congo haemorrhagic fever*" OR CCHF OR "congo virus*" OR "Crimean hemorrhagic fever*" OR Marburg OR MVD OR "Middle East Respiratory syndrome coronavirus" OR "MERS-CoV" OR "MERS Virus*" OR "Middle East respiratory syndrome" OR Merbecovirus*))  AND  TITLE-ABS-KEY ("risk assessment*" OR "risk analys*" OR "risk manag*" OR "risk communication*" OR "joint assessment" OR "risk mitigation" OR "mitigating risk*" OR "decision making" OR "assessment framework*" OR "analysis framework*" OR mitigation OR "integrated risk*" OR (risks W/3 hazard*))  AND  TITLE-ABS-KEY (("one health*" OR tripartite* OR "transdisciplinary health*" OR "trans disciplinary health*" OR "integrated health*") OR (((human* W/3 (animal* OR agricultur* OR livestock*))) AND (environment* OR ecosystem* OR "planetary health")))  AND  TITLE-ABS-KEY (disaster* OR postdisaster* OR posthazard* OR hazard* OR avalanche* OR "cyclonic storm*" OR cyclone* OR hurricane* OR "tropical storm*" OR typhoon* OR drought* OR earthquake* OR flood* OR landslide* OR rockslide* OR mudslide* OR "earth tide*" OR "ocean tide*" OR tidalwave* OR "tidal wave*" OR tsunami* OR tornado* OR wildfire* OR "wildland fire*" OR "brush fire*" OR "forest fire*" OR "wild fire*" OR "facility fire*" OR volcanic* OR "major rainfall*" OR storm OR storms OR fire*  OR volcano* OR volcanic OR "extreme temperature*" OR "extreme weather*" OR heatwave* OR "heat wave*" OR "extreme heat*" OR "extreme cold*" OR rainfall OR humidity OR altitude OR climate* OR climatic OR "global warm*" OR "greenhouse effect*" OR "greenhouse gas" OR "green house effect*" OR "green house gas" OR "green house gases" OR "greenhouse gases" OR "ozone depletion" OR "ozone hole*" OR "carbon foot*" OR deforestation OR reforestation OR logging OR conservation OR "carrying capacit*" OR "environmental protection" OR "environmental biodegradation" OR "sustainable development*" OR "smart growth" OR Meteorological OR Anthropogenic OR "human impact*" OR "land degradation" OR "forest clearing" OR "forest restoration" OR "carbon sink*" OR "ecosystem respiration" OR "carbon flux*" OR environment* OR livestock* OR temperature* OR warm* OR meteo* OR ecology OR ecologically OR ecological OR ecosystem* OR biodiversity OR "land use" OR wilderness OR wildlife OR forest*)  AND  ( PUBYEAR > 1999 ) AND NOT ( INDEX ( medline ) OR INDEX ( embase ) )  **Web of Science**  TS= ((Zoono* OR epidemic* OR "disease transmission" OR "disease spread" OR "disease cluster*" OR "cluster of cases" OR (animal* NEAR/5 human*) OR ("infectious disease*" NEAR/5 outbreak*) OR (emerging NEAR/5 disease*) OR (reemerging NEAR/5 disease*) OR spillover* OR animal* OR livestock* OR (viral AND ("biological threat*" OR "bio-threat*")) OR "Crimean-congo hemorrhagic fever*" OR "Crimean-Congo haemorrhagic fever*" OR CCHF OR "congo virus*" OR "Crimean hemorrhagic fever*" OR Marburg OR MVD OR "Middle East Respiratory syndrome coronavirus" OR "MERS-CoV" OR "MERS Virus*" OR "Middle East respiratory syndrome" OR Merbecovirus*))  AND  TS= ("risk assessment*" OR "risk analys*" OR "risk manag*" OR "risk communication*" OR "joint assessment" OR "risk mitigation" OR "mitigating risk*" OR "decision making" OR "assessment framework*" OR "analysis framework*" OR mitigation OR "integrated risk*" OR (risks NEAR/3 hazard*))  AND  TS= (("one health*" OR tripartite* OR "transdisciplinary health*" OR "trans disciplinary health*" OR "integrated health*") OR (((human* NEAR/3 (animal* OR agricultur* OR livestock*))) AND (environment* OR ecosystem* OR "planetary health")))  AND  TS= (disaster* OR postdisaster* OR posthazard* OR hazard* OR avalanche* OR "cyclonic storm*" OR cyclone* OR hurricane* OR "tropical storm*" OR typhoon* OR drought* OR earthquake* OR flood* OR landslide* OR rockslide* OR mudslide* OR "earth tide*" OR "ocean tide*" OR tidalwave* OR "tidal wave*" OR tsunami* OR tornado* OR wildfire* OR "wildland fire*" OR "brush fire*" OR "forest fire*" OR "wild fire*" OR "facility fire*" OR volcanic* OR "major rainfall*" OR storm OR storms OR fire* OR volcano* OR volcanic OR "extreme temperature*" OR "extreme weather*" OR heatwave* OR "heat wave*" OR "extreme heat*" OR "extreme cold*" OR rainfall OR humidity OR altitude OR climate* OR climatic OR "global warm*" OR "greenhouse effect*" OR "greenhouse gas" OR "green house effect*" OR "green house gas" OR "green house gases" OR "greenhouse gases" OR "ozone depletion" OR "ozone hole*" OR "carbon foot*" OR deforestation OR reforestation OR logging OR conservation OR "carrying capacit*" OR "environmental protection" OR "environmental biodegradation" OR "sustainable development*" OR "smart growth" OR Meteorological OR Anthropogenic OR "human impact*" OR "land degradation" OR "forest clearing" OR "forest restoration" OR "carbon sink*" OR "ecosystem respiration" OR "carbon flux*" OR environment* OR livestock* OR temperature* OR warm* OR meteo* OR ecology OR ecologically OR ecological OR ecosystem* OR biodiversity OR "land use" OR wilderness OR wildlife OR forest*)  **CABI Digital Library**  Title:(Zoono* OR epidemic* OR "disease transmission" OR "disease spread" OR "disease cluster" OR "disease clusters" OR "cluster of cases" OR "animal human" OR "animals human" OR "animal humans" OR "animals humans" OR "infectious disease outbreak" OR "infectious disease outbreaks" OR "infectious diseases outbreak" OR "infectious diseases outbreaks" OR "emerging disease" OR "emerging diseases" OR "reemerging disease" OR "reemerging diseases" OR spillover* OR animal* OR livestock* OR "Crimean-congo hemorrhagic fever" OR "Crimean-congo hemorrhagic fevers" OR "Crimean-Congo haemorrhagic fever" OR "Crimean-Congo haemorrhagic fevers" OR CCHF OR "congo virus" OR "congo viruses" OR "Crimean hemorrhagic fever" OR "Crimean hemorrhagic fevers" OR Marburg OR MVD OR "Middle East Respiratory syndrome coronavirus" OR "MERS-CoV" OR "MERS Virus" OR "MERS Viruses" OR "Middle East respiratory syndrome" OR Merbecovirus*) OR Title:(viral AND ("biological threat" OR "biological threats" OR "bio-threat" OR "bio-threats")) OR ab:(Zoono* OR epidemic* OR "disease transmission" OR "disease spread" OR "disease cluster" OR "disease clusters" OR "cluster of cases" OR "animal human" OR "animals human" OR "animal humans" OR "animals humans" OR "infectious disease outbreak" OR "infectious disease outbreaks" OR "infectious diseases outbreak" OR "infectious diseases outbreaks" OR "emerging disease" OR "emerging diseases" OR "reemerging disease" OR "reemerging diseases" OR spillover* OR animal* OR livestock* OR "Crimean-congo hemorrhagic fever" OR "Crimean-congo hemorrhagic fevers" OR "Crimean-Congo haemorrhagic fever" OR "Crimean-Congo haemorrhagic fevers" OR CCHF OR "congo virus" OR "congo viruses" OR "Crimean hemorrhagic fever" OR "Crimean hemorrhagic fevers" OR Marburg OR MVD OR "Middle East Respiratory syndrome coronavirus" OR "MERS-CoV" OR "MERS Virus" OR "MERS Viruses" OR "Middle East respiratory syndrome" OR Merbecovirus*) OR ab:(viral AND ("biological threat" OR "biological threats" OR "bio-threat" OR "bio-threats")) OR indexingterm:(Zoono* OR epidemic* OR "disease transmission" OR "disease spread" OR "disease cluster" OR "disease clusters" OR "cluster of cases" OR "animal human" OR "animals human" OR "animal humans" OR "animals humans" OR "infectious disease outbreak" OR "infectious disease outbreaks" OR "infectious diseases outbreak" OR "infectious diseases outbreaks" OR "emerging disease" OR "emerging diseases" OR "reemerging disease" OR "reemerging diseases" OR spillover* OR animal* OR livestock* OR "Crimean-congo hemorrhagic fever" OR "Crimean-congo hemorrhagic fevers" OR "Crimean-Congo haemorrhagic fever" OR "Crimean-Congo haemorrhagic fevers" OR CCHF OR "congo virus" OR "congo viruses" OR "Crimean hemorrhagic fever" OR "Crimean hemorrhagic fevers" OR Marburg OR MVD OR "Middle East Respiratory syndrome coronavirus" OR "MERS-CoV" OR "MERS Virus" OR "MERS Viruses" OR "Middle East respiratory syndrome" OR Merbecovirus*) OR indexing:(viral AND ("biological threat" OR "biological threats" OR "bio-threat" OR "bio-threats"))  AND  Title:("risk assessment" OR "risk assessments" OR "risk analysis" OR "risk analyses" OR "risk management" OR "risk communication" OR "joint assessment" OR "risk mitigation" OR "mitigating risk" OR "mitigating risks" OR "decision making" OR "assessment framework" OR "assessment frameworks" OR "analysis framework" OR "analysis frameworks" OR mitigation OR "integrated risk" OR "integrated risks" OR "risk hazard" OR "risk hazards" OR "risks hazard" OR "risks hazards") OR ab:("risk assessment" OR "risk assessments" OR "risk analysis" OR "risk analyses" OR "risk management" OR "risk communication" OR "joint assessment" OR "risk mitigation" OR "mitigating risk" OR "mitigating risks" OR "decision making" OR "assessment framework" OR "assessment frameworks" OR "analysis framework" OR "analysis frameworks" OR mitigation OR "integrated risk" OR "integrated risks" OR "risk hazard" OR "risk hazards" OR "risks hazard" OR "risks hazards") OR indexingterm:("risk assessment" OR "risk assessments" OR "risk analysis" OR "risk analyses" OR "risk management" OR "risk communication" OR "joint assessment" OR "risk mitigation" OR "mitigating risk" OR "mitigating risks" OR "decision making" OR "assessment framework" OR "assessment frameworks" OR "analysis framework" OR "analysis frameworks" OR mitigation OR "integrated risk" OR "integrated risks" OR "risk hazard" OR "risk hazards" OR "risks hazard" OR "risks hazards")  AND  Title:("one health" OR "one healthy" OR tripartite* OR "transdisciplinary health" OR "transdisciplinary healthy" OR "trans disciplinary health" OR "trans disciplinary healthy" OR "integrated health" OR "integrated healthy") OR title:(((human* AND (animal* OR agricultur* OR livestock*))) AND (environment* OR ecosystem* OR "planetary health")) OR ab:("one health" OR "one healthy" OR tripartite* OR "transdisciplinary health" OR "transdisciplinary healthy" OR "trans disciplinary health" OR "trans disciplinary healthy" OR "integrated health" OR "integrated healthy") OR ab:(((human* AND (animal* OR agricultur* OR livestock*))) AND (environment* OR ecosystem* OR "planetary health")) OR indexingterm:("one health" OR "one healthy" OR tripartite* OR "transdisciplinary health" OR "transdisciplinary healthy" OR "trans disciplinary health" OR "trans disciplinary healthy" OR "integrated health" OR "integrated healthy") OR indexingterm:(((human* AND (animal* OR agricultur* OR livestock*))) AND (environment* OR ecosystem* OR "planetary health"))  AND  Title:(disaster* OR postdisaster* OR posthazard* OR hazard* OR avalanche* OR cyclone* OR hurricane* OR typhoon* OR drought* OR earthquake* OR flood* OR landslide* OR rockslide* OR mudslide* OR "earth tide" OR "earth tides" OR "ocean tide" OR "ocean tides" OR tidalwave* OR "tidal wave" OR "tidal waves" OR tsunami* OR tornado* OR wildfire* OR volcanic* OR "major rainfall" OR "major rainfalls" OR storm OR storms OR fire* OR volcano* OR "extreme temperature" OR "extreme temperatures" OR "extreme weather" OR "extreme weathers" OR heatwave* OR "heat wave" OR "heat waves" OR "extreme heat" OR "extreme heats" OR "extreme cold" OR rainfall OR humidity OR altitude OR climate* OR climatic OR "global warm" OR "global warming" OR "greenhouse effect" OR "greenhouse effects" OR "greenhouse gas" OR "green house effect" OR "green house effects" OR "green house gas" OR "green house gases" OR "greenhouse gases" OR "ozone depletion" OR "ozone hole" OR "ozone holes" OR "carbon foot" OR "carbon footprint" OR "carbon footprints" OR deforestation OR reforestation OR logging OR conservation OR "carrying capacity" OR "carrying capacities" OR "environmental protection" OR "environmental biodegradation" OR "sustainable development" OR "sustainable developments" OR "smart growth" OR Meteorological OR Anthropogenic OR "human impact" OR "human impacts" OR "land degradation" OR "forest clearing" OR "forest restoration" OR "carbon sink" OR "carbon sinks" OR "carbon sinking" OR "ecosystem respiration" OR "carbon flux" OR environment* OR livestock* OR temperature* OR warm* OR meteo* OR ecology OR ecologically OR ecological OR ecosystem* OR biodiversity OR "land use" OR wilderness OR wildlife OR forest*) OR ab:(disaster* OR postdisaster* OR posthazard* OR hazard* OR avalanche* OR cyclone* OR hurricane* OR typhoon* OR drought* OR earthquake* OR flood* OR landslide* OR rockslide* OR mudslide* OR "earth tide" OR "earth tides" OR "ocean tide" OR "ocean tides" OR tidalwave* OR "tidal wave" OR "tidal waves" OR tsunami* OR tornado* OR wildfire* OR volcanic* OR "major rainfall" OR "major rainfalls" OR storm OR storms OR fire* OR volcano* OR "extreme temperature" OR "extreme temperatures" OR "extreme weather" OR "extreme weathers" OR heatwave* OR "heat wave" OR "heat waves" OR "extreme heat" OR "extreme heats" OR "extreme cold" OR rainfall OR humidity OR altitude OR climate* OR climatic OR "global warm" OR "global warming" OR "greenhouse effect" OR "greenhouse effects" OR "greenhouse gas" OR "green house effect" OR "green house effects" OR "green house gas" OR "green house gases" OR "greenhouse gases" OR "ozone depletion" OR "ozone hole" OR "ozone holes" OR "carbon foot" OR "carbon footprint" OR "carbon footprints" OR deforestation OR reforestation OR logging OR conservation OR "carrying capacity" OR "carrying capacities" OR "environmental protection" OR "environmental biodegradation" OR "sustainable development" OR "sustainable developments" OR "smart growth" OR Meteorological OR Anthropogenic OR "human impact" OR "human impacts" OR "land degradation" OR "forest clearing" OR "forest restoration" OR "carbon sink" OR "carbon sinks" OR "carbon sinking" OR "ecosystem respiration" OR "carbon flux" OR environment* OR livestock* OR temperature* OR warm* OR meteo* OR ecology OR ecologically OR ecological OR ecosystem* OR biodiversity OR "land use" OR wilderness OR wildlife OR forest*) OR indexingterm:(disaster* OR postdisaster* OR posthazard* OR hazard* OR avalanche* OR cyclone* OR hurricane* OR typhoon* OR drought* OR earthquake* OR flood* OR landslide* OR rockslide* OR mudslide* OR "earth tide" OR "earth tides" OR "ocean tide" OR "ocean tides" OR tidalwave* OR "tidal wave" OR "tidal waves" OR tsunami* OR tornado* OR wildfire* OR volcanic* OR "major rainfall" OR "major rainfalls" OR storm OR storms OR fire* OR volcano* OR "extreme temperature" OR "extreme temperatures" OR "extreme weather" OR "extreme weathers" OR heatwave* OR "heat wave" OR "heat waves" OR "extreme heat" OR "extreme heats" OR "extreme cold" OR rainfall OR humidity OR altitude OR climate* OR climatic OR "global warm" OR "global warming" OR "greenhouse effect" OR "greenhouse effects" OR "greenhouse gas" OR "green house effect" OR "green house effects" OR "green house gas" OR "green house gases" OR "greenhouse gases" OR "ozone depletion" OR "ozone hole" OR "ozone holes" OR "carbon foot" OR "carbon footprint" OR "carbon footprints" OR deforestation OR reforestation OR logging OR conservation OR "carrying capacity" OR "carrying capacities" OR "environmental protection" OR "environmental biodegradation" OR "sustainable development" OR "sustainable developments" OR "smart growth" OR Meteorological OR Anthropogenic OR "human impact" OR "human impacts" OR "land degradation" OR "forest clearing" OR "forest restoration" OR "carbon sink" OR "carbon sinks" OR "carbon sinking" OR "ecosystem respiration" OR "carbon flux" OR environment* OR livestock* OR temperature* OR warm* OR meteo* OR ecology OR ecologically OR ecological OR ecosystem* OR biodiversity OR "land use" OR wilderness OR wildlife OR forest*)  CAB Direct  (Zoono* OR epidemic* OR "disease transmission" OR "disease spread" OR "disease cluster*" OR "cluster of cases" OR "animal* human*" OR "infectious disease* outbreak*" OR "emerging disease*" OR "reemerging disease*" OR spillover* OR animal* OR livestock* OR (viral AND ("biological threat*" OR "bio-threat*")) OR "Crimean-congo hemorrhagic fever*" OR "Crimean-Congo haemorrhagic fever*" OR CCHF OR "congo virus*" OR "Crimean hemorrhagic fever*" OR Marburg OR MVD OR "Middle East Respiratory syndrome coronavirus" OR "MERS-CoV" OR "MERS Virus*" OR "Middle East respiratory syndrome" OR Merbecovirus*)  AND  ("risk assessment*" OR "risk analys*" OR "risk manag*" OR "risk communication*" OR "joint assessment" OR "risk mitigation" OR "mitigating risk*" OR "decision making" OR "assessment framework*" OR "analysis framework*" OR mitigation OR "integrated risk*" OR "risk* hazard*")  AND  ("one health*" OR tripartite* OR "transdisciplinary health*" OR "trans disciplinary health*" OR "integrated health*") OR (((human* AND (animal* OR agricultur* OR livestock*))) AND (environment* OR ecosystem* OR "planetary health"))  AND  (disaster* OR postdisaster* OR posthazard* OR hazard* OR avalanche* OR cyclone* OR hurricane* OR typhoon* OR drought* OR earthquake* OR flood* OR landslide* OR rockslide* OR mudslide* OR "earth tide*" OR "ocean tide*" OR tidalwave* OR "tidal wave*" OR tsunami* OR tornado* OR wildfire* OR volcanic* OR "major rainfall*" OR storm OR storms OR fire* OR volcano* OR "extreme temperature*" OR "extreme weather*" OR heatwave* OR "heat wave*" OR "extreme heat*" OR "extreme cold*" OR rainfall OR humidity OR altitude OR climate* OR climatic OR "global warm*" OR "greenhouse effect*" OR "greenhouse gas" OR "green house effect*" OR "green house gas" OR "green house gases" OR "greenhouse gases" OR "ozone depletion" OR "ozone hole*" OR "carbon foot*" OR deforestation OR reforestation OR logging OR conservation OR "carrying capacit*" OR "environmental protection" OR "environmental biodegradation" OR "sustainable development*" OR "smart growth" OR Meteorological OR Anthropogenic OR "human impact*" OR "land degradation" OR "forest clearing" OR "forest restoration" OR "carbon sink*" OR "ecosystem respiration" OR "carbon flux*" OR environment* OR livestock* OR temperature* OR warm* OR meteo* OR ecology OR ecologically OR ecological OR ecosystem* OR biodiversity OR "land use" OR wilderness OR wildlife OR forest*) |
| 1. **What types of policy recommendations applicable to the East Africa region are in place to address environmental drivers of emerging/reemerging zoonotic biological threats such as CCHFV, MARV, and MERS-CoV?**   Inclusion criteria   - Policy recommendations - Recommendations could apply to East Africa - English Language | **Pubmed**  **("Zoonoses"[Mesh] OR "Disease Outbreaks"[Mesh:NoExp] OR "Disease Hotspot"[Mesh] OR "Epidemics"[Mesh:NoExp] OR "Space-Time Clustering"[Mesh] OR "Disease Transmission, Infectious"[Mesh]** OR**"Animals"[Mesh] OR "Livestock"[Mesh]** **OR "Communicable Diseases, Emerging"[Mesh] OR "Communicable Diseases"[Mesh]** OR Zoono*[tw] OR "animal to human"[tiab:~5] OR "animal to humans"[tiab:~5] OR "animals to human"[tiab:~5] OR "animals to humans"[tiab:~5] OR "infectious disease outbreak"[tiab:~5] OR "infectious disease outbreaks"[tiab:~5] OR "infectious diseases outbreak"[tiab:~5] OR "infectious diseases outbreaks"[tiab:~5] OR epidemic*[tw] OR "disease transmission"[tw] OR "disease spread"[tw] OR "disease cluster*"[tw] OR "cluster of cases"[tw] OR "emerging diseases"[tiab:~5] OR "reemerging diseases"[tiab:~5]  OR "re emerging diseases"[tiab:~5] OR spillover*[tw] OR animal*[tw] OR livestock*[tw] OR (viral[tw] AND ("biological threat*"[tw] OR "bio-threat*"[tw])) OR **"Hemorrhagic Fever Virus, Crimean-Congo"[Mesh]** OR "Crimean-congo hemorrhagic fever*"[tw] OR CCHF[tw] OR "congo virus*"[tw] OR "Crimean hemorrhagic fever*"[tw] OR **"Marburg Virus Disease"[Mesh]** OR Marburg[tw] OR MVD[tw] OR **"Middle East Respiratory Syndrome Coronavirus"[Mesh]** OR "Middle East Respiratory syndrome coronavirus"[tw] OR "MERS-CoV"[tw] OR "MERS Virus*"[tw] OR "Middle East respiratory syndrome"[tw] OR Merbecovirus*[tw])  **("Africa, Eastern"[Mesh]** OR "Africa east"[tiab:~2] OR "Africa eastern"[tiab:~2] OR "African east"[tiab:~2] OR "African eastern"[tiab:~2] OR "africans east"[tiab:~2] OR "africans eastern"[tiab:~2] OR Burundi*[tw] OR Comoros*[tw] OR Djibouti*[tw] OR Eritrea*[tw] OR Ethiopia*[tw] OR Kenya*[tw] OR Madagascar*[tw] OR Mauritius* [tw] OR Rwanda*[tw] OR Seychelles*[tw] OR Somalia*[tw] OR Sudan*[tw] OR Tanzania*[tw] OR Uganda*[tw] OR "Addis Ababa"[tw] OR Antananarivo[tw] OR Asmara[tw] OR Bujumbura[tw] OR "Dar es Salaam"[tw] OR "Dire Dawa"[tw] OR "Djibouti City"[tw] OR Dodoma[tw] OR Gitega[tw] OR Hargeisa[tw] OR Juba[tw] OR Kampala[tw] OR Keren[tw] OR Khartoum[tw] OR Kigali[tw] OR Kisumu[tw] OR Lindi[tw] OR Mahajanga[tw] OR Mbarara[tw] OR Mogadishu[tw] OR Mombasa[tw] OR Muhanga[tw] OR Moroni[tw] OR Muyinga[tw] OR Mwanza[tw] OR Nairobi[tw] OR Ngozi[tw] OR Omdurman[tw] OR "Port Louis"[tw] OR Victoria[tw] OR "Great Lakes Region*"[tw] OR "Horn of Africa*"[tw] OR "Nile Valle*"[tw])  AND  **("Climate"[Mesh] OR "Ecosystem"[Mesh] OR "Natural Resources"[Mesh] OR "Disasters"[Mesh:NoExp] OR "Disaster Planning"[Mesh:NoExp] OR "Natural Disasters"[Mesh] OR "Environmental Health"[Mesh:NoExp] OR "Sanitation"[Mesh:NoExp] OR "Conservation of Natural Resources"[Mesh] OR "Wastewater"[Mesh] OR "Waste Management"[Mesh:NoExp] OR** ecosystem*[tw] OR ecological*[tw] OR climate*[tw] OR "natural resources"[tw] OR "disaster plan*"[tw] OR "natural disaster*"[tw] OR "environmental health"[tw] OR "environmental protection"[tw] **) OR** (**("Environment"[Mesh] OR** environment*[tw]) AND (drivers[tw] OR factors[tw]))  AND  (**"Policy"[Mesh:NoExp] OR "Public Policy"[Mesh:NoExp] OR "Environmental Policy"[Mesh:NoExp] OR "Health Policy"[Mesh:NoExp] OR "International Health Regulations"[Mesh] OR "Sustainable Development"[Mesh] OR "Policy Making"[Mesh:NoExp] OR "Government Regulation"[Mesh] OR "Legislation as Topic"[Mesh:NoExp]** **OR "Decision Making, Shared"[Mesh] OR "Stakeholder Participation"[Mesh] OR "Health Planning Guidelines"[Mesh] OR "Health Priorities"[Mesh] OR "Health Planning"[Mesh:NoExp]**OR Policy change*[tw] OR Policy problem*[tw] OR Policy forecast*[tw] OR Policy solution*[tw] OR Policy stud*[tw] OR Policy environment*[tw] OR Policy development*[tw] OR Policy option*[tw] OR Policy alternative*[tw] OR Policy recommendation*[tw] OR policy implement*[tw] OR Policy maker*[tw] OR Decision maker*[tw] OR "National government*"[tw] OR Policy adoption[tw] OR Policy evaluation*[tw] OR Policy outcome*[tw] OR Policy performance[tw] OR "Health policy and planning"[tw] OR "policy analysis"[tw] OR "Policy formulation*"[tw] OR Policy brief*[tw] OR Policy reform*[tw] OR Public policy[tw] OR public policies[tw] OR Stakeholder engagement*[tw] OR Health polic*[tw] OR "Evidence-based policy"[tw] OR Evidence-based policies*[tw] OR "Evidence-informed policy"[tw] OR "Evidence-informed policies"[tw] OR "cross sectoral decision making"[tiab:~5] OR "risk informed decision making"[tw] OR "government authorit*"[tw] OR "health planning"[tw] OR "Health care reform*"[tw] OR Regulation*[tw] OR "Land management"[tw] OR "Land policy"[tw] OR "land policies"[tw] OR "Policy engagement"[tw] OR "Policy environment*"[tw])  **Embase**  **('zoonosis'/exp OR 'epidemic'/de OR 'disease hotspot'/exp OR 'spatiotemporal analysis'/exp OR 'zoonotic transmission'/exp OR 'pathogen transmission'/exp OR 'nosocomial transmission'/exp OR 'community transmission'/exp OR 'animal'/exp OR 'livestock'/exp OR 'communicable disease'/exp OR 'emerging infectious disease'/exp OR 'Marburg hemorrhagic fever'/exp OR 'Crimean-Congo hemorrhagic fever virus'/exp OR 'Middle East respiratory syndrome coronavirus'/exp) OR** (Zoono* OR epidemic* OR "disease transmission" OR "disease spread" OR "disease cluster*" OR "cluster of cases" OR (animal* NEAR/5 human*) OR ("infectious disease*" NEAR/5 outbreak*) OR (emerging NEAR/5 disease*) OR (reemerging NEAR/5 disease*) OR spillover* OR animal* OR livestock* OR (viral AND ("biological threat*" OR "bio-threat*")) OR "Crimean-congo hemorrhagic fever*" OR CCHF OR "congo virus*" OR "Crimean hemorrhagic fever*" OR Marburg OR MVD OR "Middle East Respiratory syndrome coronavirus" OR "MERS-CoV" OR "MERS Virus*" OR "Middle East respiratory syndrome" OR Merbecovirus*):ab,ti,kw  AND  **'east african'/exp** OR ((Africa* NEAR/2 east*) OR Burundi* OR Comoros* OR Djibouti* OR Eritrea* OR Ethiopia* OR Kenya* OR Madagascar* OR Mauritius* OR Rwanda* OR Seychelles* OR Somalia* OR Sudan* OR Tanzania* OR Uganda* OR "Addis Ababa" OR Antananarivo OR Asmara OR Bujumbura OR "Dar es Salaam" OR "Dire Dawa" OR "Djibouti City" OR Dodoma OR Gitega OR Hargeisa OR Juba OR Kampala OR Keren OR Khartoum OR Kigali OR Kisumu OR Lindi OR Mahajanga OR Mbarara OR Mogadishu OR Mombasa OR Muhanga OR Moroni OR Muyinga OR Mwanza OR Nairobi OR Ngozi OR Omdurman OR "Port Louis" OR Victoria OR "Great Lakes Region*" OR "Horn of Africa*" OR "Nile Valle*"):ab,ti,kw  AND  **('climate'/exp OR 'ecosystem'/exp OR 'natural resource'/exp OR 'disaster'/de OR 'natural disaster'/exp OR 'disaster planning'/de OR 'disaster mitigation'/exp OR 'disaster preparedness'/exp OR 'environmental health'/de OR 'sanitation'/de OR 'pollution control'/exp OR 'environmental protection'/exp OR 'waste management'/de OR 'sewage treatment'/exp)** OR ("environmental health" OR "environmental protection" OR ecosystem* OR ecological* OR climate* OR "natural resources" OR "disaster plan*" OR "natural disaster*"):ab,ti,kw OR ((**'environment'/exp**) AND (drivers OR factors):ab,ti,kw) OR ((environment*:ab,ti,kw) AND (drivers OR factors)):ab,ti,kw  AND  **('policy'/de OR 'public policy'/de OR 'environmental policy'/de OR 'health care policy'/de OR 'international health regulation'/exp OR 'sustainable development'/exp OR 'management'/de OR 'government regulation'/exp OR 'law'/de OR 'shared decision making'/exp OR 'stakeholder engagement'/exp OR 'health care planning'/exp)** OR ("Policy change*" OR "Policy problem*" OR "Policy forecast*" OR "Policy solution*" OR "Policy stud*" OR "Policy environment*" OR "Policy development*" OR "Policy option*" OR "Policy alternative*" OR "Policy recommendation*" OR "policy implement*" OR "Policy maker*" OR "Decision maker*" OR "National government*" OR "Policy adoption" OR "Policy evaluation*" OR "Policy outcome*" OR "Policy performance" OR "Health policy and planning" OR "policy analysis" OR "Policy formulation*" OR "Policy brief*" OR "Policy reform*" OR "Public policy" OR "public policies" OR "Stakeholder engagement*" OR "Health polic*" OR "Evidence-based policy" OR "Evidence-based policies*" OR "Evidence-informed policy" OR "Evidence-informed policies" OR ("cross sectoral" NEAR/5 "decision making") OR "risk informed decision making" OR "government authorit*" OR "health planning" OR "Health care reform*" OR Regulation* OR "Land management" OR "Land policy" OR "land policies" OR "Policy engagement" OR "Policy environment*"):ab,ti,kw  AND  [2000-2023]/py  **Scopus**  TITLE-ABS-KEY((Zoono* OR epidemic* OR "disease transmission" OR "disease spread" OR "disease cluster*" OR "cluster of cases" OR (animal* W/5 human*) OR ("infectious disease*" W/5 outbreak*) OR (emerging W/5 disease*) OR (reemerging W/5 disease*) OR spillover* OR animal* OR livestock* OR (viral AND ("biological threat*" OR "bio-threat*")) OR "Crimean-congo hemorrhagic fever*" OR CCHF OR "congo virus*" OR "Crimean hemorrhagic fever*" OR Marburg OR MVD OR "Middle East Respiratory syndrome coronavirus" OR "MERS-CoV" OR "MERS Virus*" OR "Middle East respiratory syndrome" OR Merbecovirus*))  AND  TITLE-ABS-KEY ((Africa* W/2 east*) OR Burundi* OR Comoros* OR Djibouti* OR Eritrea* OR Ethiopia* OR Kenya* OR Madagascar* OR Mauritius* OR Rwanda* OR Seychelles* OR Somalia* OR Sudan* OR Tanzania* OR Uganda* OR "Addis Ababa" OR Antananarivo OR Asmara OR Bujumbura OR "Dar es Salaam" OR "Dire Dawa" OR "Djibouti City" OR Dodoma OR Gitega OR Hargeisa OR Juba OR Kampala OR Keren OR Khartoum OR Kigali OR Kisumu OR Lindi OR Mahajanga OR Mbarara OR Mogadishu OR Mombasa OR Muhanga OR Moroni OR Muyinga OR Mwanza OR Nairobi OR Ngozi OR Omdurman OR "Port Louis" OR Victoria OR "Great Lakes Region*" OR "Horn of Africa*" OR "Nile Valle*")  AND  TITLE-ABS-KEY (ecosystem* OR ecological* OR climate* OR "natural resources" OR "disaster plan*" OR "natural disaster*" OR "environmental health" OR "environmental protection") OR TITLE-ABS-KEY(environment* AND (drivers OR factors))  AND  TITLE-ABS-KEY ("Policy change*" OR "Policy problem*" OR "Policy forecast*" OR "Policy solution*" OR "Policy stud*" OR "Policy environment*" OR "Policy development*" OR "Policy option*" OR "Policy alternative*" OR "Policy recommendation*" OR "policy implement*" OR "Policy maker*" OR "Decision maker*" OR "National government*" OR "Policy adoption" OR "Policy evaluation*" OR "Policy outcome*" OR "Policy performance" OR "Health policy and planning" OR "policy analysis" OR "Policy formulation*" OR "Policy brief*" OR "Policy reform*" OR "Public policy" OR "public policies" OR "Stakeholder engagement*" OR "Health polic*" OR "Evidence-based policy" OR "Evidence-based policies*" OR "Evidence-informed policy" OR "Evidence-informed policies" OR ("cross sectoral" W/5 "decision making") OR "risk informed decision making" OR "government authorit*" OR "health planning" OR "Health care reform*" OR Regulation* OR "Land management" OR "Land policy" OR "land policies" OR "Policy engagement" OR "Policy environment*")  AND  ( PUBYEAR > 1999 ) AND NOT ( INDEX ( medline ) OR INDEX ( embase ) )  **Web of Science**  TS= ((Zoono* OR epidemic* OR "disease transmission" OR "disease spread" OR "disease cluster*" OR "cluster of cases" OR (animal* NEAR/5 human*) OR ("infectious disease*" NEAR/5 outbreak*) OR (emerging NEAR/5 disease*) OR (reemerging NEAR/5 disease*) OR spillover* OR animal* OR livestock* OR (viral AND ("biological threat*" OR "bio-threat*")) OR "Crimean-congo hemorrhagic fever*" OR CCHF OR "congo virus*" OR "Crimean hemorrhagic fever*" OR Marburg OR MVD OR "Middle East Respiratory syndrome coronavirus" OR "MERS-CoV" OR "MERS Virus*" OR "Middle East respiratory syndrome" OR Merbecovirus*))  AND  TS= ((Zoono* OR epidemic* OR "disease transmission" OR "disease spread" OR "disease cluster*" OR "cluster of cases" OR (animal* NEAR/5 human*) OR ("infectious disease*" NEAR/5 outbreak*) OR (emerging NEAR/5 disease*) OR (reemerging NEAR/5 disease*) OR spillover* OR animal* OR livestock* OR (viral AND ("biological threat*" OR "bio-threat*")) OR "Crimean-congo hemorrhagic fever*" OR CCHF OR "congo virus*" OR "Crimean hemorrhagic fever*" OR Marburg OR MVD OR "Middle East Respiratory syndrome coronavirus" OR "MERS-CoV" OR "MERS Virus*" OR "Middle East respiratory syndrome" OR Merbecovirus*))  AND  TS=((Africa* NEAR/2 east*) OR Burundi* OR Comoros* OR Djibouti* OR Eritrea* OR Ethiopia* OR Kenya* OR Madagascar* OR Mauritius* OR Rwanda* OR Seychelles* OR Somalia* OR Sudan* OR Tanzania* OR Uganda* OR "Addis Ababa" OR Antananarivo OR Asmara OR Bujumbura OR "Dar es Salaam" OR "Dire Dawa" OR "Djibouti City" OR Dodoma OR Gitega OR Hargeisa OR Juba OR Kampala OR Keren OR Khartoum OR Kigali OR Kisumu OR Lindi OR Mahajanga OR Mbarara OR Mogadishu OR Mombasa OR Muhanga OR Moroni OR Muyinga OR Mwanza OR Nairobi OR Ngozi OR Omdurman OR "Port Louis" OR Victoria OR "Great Lakes Region*" OR "Horn of Africa*" OR "Nile Valle*")  AND  TS= (environment* OR ecosystem* OR ecological* OR climate* OR "natural resources" OR "disaster plan*" OR "natural disaster*" OR "environmental health" OR "environmental protection") OR TS=(environment* AND (drivers OR factors))  AND  TS= ("Policy change*" OR "Policy problem*" OR "Policy forecast*" OR "Policy solution*" OR "Policy stud*" OR "Policy environment*" OR "Policy development*" OR "Policy option*" OR "Policy alternative*" OR "Policy recommendation*" OR "policy implement*" OR "Policy maker*" OR "Decision maker*" OR "National government*" OR "Policy adoption" OR "Policy evaluation*" OR "Policy outcome*" OR "Policy performance" OR "Health policy and planning" OR "policy analysis" OR "Policy formulation*" OR "Policy brief*" OR "Policy reform*" OR "Public policy" OR "public policies" OR "Stakeholder engagement*" OR "Health polic*" OR "Evidence-based policy" OR "Evidence-based policies*" OR "Evidence-informed policy" OR "Evidence-informed policies" OR ("cross sectoral" NEAR/5 "decision making") OR "risk informed decision making" OR "government authorit*" OR "health planning" OR "Health care reform*" OR Regulation* OR "Land management" OR "Land policy" OR "land policies" OR "Policy engagement" OR "Policy environment*")  AND  PY=(2000 OR 2001 OR 2002 OR 2003 OR 2004 OR 2005 OR 2006 OR 2007 OR 2008 OR 2009 OR 2010 OR 2011 OR 2012 OR 2013 OR 2014 OR 2015 OR 2016 OR 2017 OR 2018 OR 2019 OR 2020 OR 2021 OR 2022 OR 2023)  **CABI Digital Library**  (Zoono* OR epidemic* OR "disease transmission" OR "disease spread" OR "disease cluster" OR "disease clusters" OR "cluster of cases" OR "animal human" OR "animals human" OR "animal humans" OR "animals humans" OR "infectious disease outbreak" OR "infectious disease outbreaks" OR "infectious diseases outbreak" OR "infectious diseases outbreaks" OR "emerging disease" OR "emerging diseases" OR "reemerging disease" OR "reemerging diseases" OR spillover* OR animal* OR livestock* OR "Crimean-congo hemorrhagic fever" OR "Crimean-congo hemorrhagic fevers" OR "Crimean-Congo haemorrhagic fever" OR "Crimean-Congo haemorrhagic fevers" OR CCHF OR "congo virus" OR "congo viruses" OR "Crimean hemorrhagic fever" OR "Crimean hemorrhagic fevers" OR Marburg OR MVD OR "Middle East Respiratory syndrome coronavirus" OR "MERS-CoV" OR "MERS Virus" OR "MERS Viruses" OR "Middle East respiratory syndrome" OR Merbecovirus*) OR (viral AND ("biological threat" OR "biological threats" OR "bio-threat" OR "bio-threats"))  AND  ("East Africa" OR "Eastern Africa" OR "East African" OR "Eastern African" OR Burundi* OR Comoros* OR Djibouti* OR Eritrea* OR Ethiopia* OR Kenya* OR Madagascar* OR Mauritius* OR Rwanda* OR Seychelles* OR Somalia* OR Sudan* OR Tanzania* OR Uganda* OR "Addis Ababa" OR Antananarivo OR Asmara OR Bujumbura OR "Dar es Salaam" OR "Dire Dawa" OR "Djibouti City" OR Dodoma OR Gitega OR Hargeisa OR Juba OR Kampala OR Keren OR Khartoum OR Kigali OR Kisumu OR Lindi OR Mahajanga OR Mbarara OR Mogadishu OR Mombasa OR Muhanga OR Moroni OR Muyinga OR Mwanza OR Nairobi OR Ngozi OR Omdurman OR "Port Louis" OR Victoria OR "Great Lakes Region" OR "Great Lakes Regions" OR "Horn of Africa" OR "Nile Valle")  AND  (ecosystem* OR ecological* OR climate* OR "natural resources" OR "disaster plan" OR "disaster plans" OR "disaster planning" OR "natural disaster" OR "natural disasters" OR "environmental health" OR "environmental protection") OR ((environment*) AND (drivers OR factors))  AND  ("Policy change" OR "Policy changes" OR "Policy problem" OR "Policy problems" OR "Policy forecast" OR "Policy forecasts" OR "Policy forecasting" OR "Policy solution" OR "Policy solutions" OR "Policy study" OR "Policy studies" OR "Policy environment" OR "Policy environments" OR "Policy development" OR "Policy developments" OR "Policy option" OR "Policy options" OR "Policy alternative" OR "Policy alternatives" OR "Policy recommendation" OR "Policy recommendations" OR "policy implementation" OR "Policy maker" OR "Policy makers" OR "Decision maker" OR "Decision makers" OR "National government" OR "National governments" OR "Policy adoption" OR "Policy evaluation" OR "Policy evaluations" OR "Policy outcome" OR "Policy outcomes" OR "Policy performance" OR "Health policy and planning" OR "policy analysis" OR "Policy formulation" OR "Policy formulations" OR "Policy brief" OR "Policy briefs" OR "Policy reform" OR "Policy reforms" OR "Public policy" OR "public policies" OR "Stakeholder engagement" OR "Stakeholder engagements" OR "Health policy" OR "Health policies" OR "Evidence-based policy" OR "Evidence-based policies" OR "Evidence-informed policy" OR "Evidence-informed policies" OR "risk informed decision making" OR "government authority" OR "government authorities" OR "health planning" OR "Health care reform" OR "Health care reforms" OR Regulation* OR "Land management" OR "Land policy" OR "land policies" OR "Policy engagement" OR "Policy environment" OR "Policy environments") OR ("cross sectoral" AND "decision making")  **CAB Direct**  (Zoono* OR epidemic* OR "disease transmission" OR "disease spread" OR "disease cluster*" OR "cluster of cases" OR "animal* human*" OR "infectious disease* outbreak*" OR "emerging disease*" OR "reemerging disease*" OR spillover* OR animal* OR livestock* OR (viral AND ("biological threat*" OR "bio-threat*")) OR ("Crimean-congo hemorrhagic fever*" OR "Crimean-Congo haemorrhagic fever*" OR CCHF OR "congo virus*" OR "Crimean hemorrhagic fever*" OR Marburg OR MVD OR "Middle East Respiratory syndrome coronavirus" OR "MERS-CoV" OR "MERS Virus*" OR "Middle East respiratory syndrome" OR Merbecovirus*)  AND  ("East* Africa*" OR "Africa* East*" OR Burundi* OR Comoros* OR Djibouti* OR Eritrea* OR Ethiopia* OR Kenya* OR Madagascar* OR Mauritius* OR Rwanda* OR Seychelles* OR Somalia* OR Sudan* OR Tanzania* OR Uganda* OR "Addis Ababa" OR Antananarivo OR Asmara OR Bujumbura OR "Dar es Salaam" OR "Dire Dawa" OR "Djibouti City" OR Dodoma OR Gitega OR Hargeisa OR Juba OR Kampala OR Keren OR Khartoum OR Kigali OR Kisumu OR Lindi OR Mahajanga OR Mbarara OR Mogadishu OR Mombasa OR Muhanga OR Moroni OR Muyinga OR Mwanza OR Nairobi OR Ngozi OR Omdurman OR "Port Louis" OR Victoria OR "Great Lakes Region*" OR "Horn of Africa*" OR "Nile Valle*")  AND  (ecosystem* OR ecological* OR climate* OR "natural resources" OR "disaster plan*" OR "natural disaster*" OR "environmental health" OR "environmental protection") OR ((environment*) AND (drivers OR factors))  AND  ("Policy change*" OR "Policy problem*" OR "Policy forecast*" OR "Policy solution*" OR "Policy stud*" OR "Policy environment*" OR "Policy development*" OR "Policy option*" OR "Policy alternative*" OR "Policy recommendation*" OR "policy implement*" OR "Policy maker*" OR "Decision maker*" OR "National government*" OR "Policy adoption" OR "Policy evaluation*" OR "Policy outcome*" OR "Policy performance" OR "Health policy and planning" OR "policy analysis" OR "Policy formulation*" OR "Policy brief*" OR "Policy reform*" OR "Public policy" OR "public policies" OR "Stakeholder engagement*" OR "Health polic*" OR "Evidence-based policy" OR "Evidence-based policies*" OR "Evidence-informed policy" OR "Evidence-informed policies" OR ("cross sectoral" AND "decision making") OR "risk informed decision making" OR "government authorit*" OR "health planning" OR "Health care reform*" OR Regulation* OR "Land management" OR "Land policy" OR "land policies" OR "Policy engagement" OR "Policy environment*") |

Supplemental Table 3 (ST3): List of articles utilized for the critical appraisal of evidence

| **Title** | **Authors** | **Published Year** | **Covidence #** | **Study** |
| --- | --- | --- | --- | --- |
| 1. **Contribution Of The MESs-CoV Research to One Health Operationalization in Ethiopia and Kenya** | Kiambi, S.; Walelign, E.; Nyariki, T.; Van 't Klooster, G.; Sitawa, R.; Kimutai, J.; Kivaria, F.; Kuria, W.; Njogu, G.; Jobre, Y.; Tewolde, N.; Gari, G.; Bebay, C.; Von Dobschuetz, S.; Gardner, E. | 2022 | #502 | Kiambi 2022 |
| 1. **Short report on implications of COVID-19 and emerging zoonotic infectious diseases for pastoralists and Africa.** | Egeru A; Dejene SW; Siya A | 2020 | #122 | Egeru 2020 |
| 1. **Dromedary Camels: Growing zoonotic disease risk at the human-livestock-wildlife interface** | Zhu, S.; Zimmerman, D.; Deem, S. | 2018 | #633 | Zhu 2018 |
| 1. **Towards a Sustainable One Health Approach to Crimean-Congo Hemorrhagic Fever Prevention: Focus Areas and Gaps in Knowledge.** | Sorvillo TE; Rodriguez SE; Hudson P; Carey M; Rodriguez LL; Spiropoulou CF; Bird BH; Spengler JR; Bente DA | 2020 | #119 | Sorvillo 2020 |
| 1. **The importance of a One Health approach for prioritizing zoonotic diseases to focus on capacity-building efforts in Uganda** | Nantima, N; Ilukor, J; Kaboyo, W; Ademun, ARO; Muwanguzi, D; Sekamatte, M; Sentumbwe, J; Monje, F; Bwire, G | 2019 | #1417 | Nantima 2019 |
| 1. **Zoonotic Pathogens of Dromedary Camels in Kenya: A Systematized Review.** | Hughes EC; Anderson NE | 2020 | #118 | Hughes 2020 |
| 1. **Uganda mountain community health system—perspectives and capacities towards emerging infectious disease surveillance** | Siya, A.; Mafigiri, R.; Migisha, R.; Kading, R.C. | 2021 | #514 | Siya 2021 |
| 1. **A descriptive study of zoonotic disease risk at the human-wildlife interface in a biodiversity hot spot in Southwestern Uganda.** | Namusisi S; Mahero M; Travis D; Pelican K; Robertson C; Mugisha L | 2021 | #101 | Namusisi 2021 |
| 1. **Lowland grazing and Marburg virus disease (MVD) outbreak in Kween district, Eastern Uganda.** | Siya A; Bazeyo W; Tuhebwe D; Tumwine G; Ezama A; Manirakiza L; Kugonza DR; Rwego IB | 2019 | #173 | Siya 2019 |
| 1. **Local, national, and regional viral haemorrhagic fever pandemic potential in Africa: a multistage analysis.** | Pigott DM; Deshpande A; Letourneau I; Morozoff C; Reiner RC Jr; Kraemer MUG; Brent SE; Bogoch II; Khan K; Biehl MH; Burstein R; Earl L; Fullman N; Messina JP; Mylne AQN; Moyes CL; Shearer FM; Bhatt S; Brady OJ; Gething PW; Weiss DJ; Tatem AJ; Caley L; De Groeve T; Vernaccini L; Golding N; Horby P; Kuhn JH; Laney SJ; Ng E; Piot P; Sankoh O; Murray CJL; Hay SI | 2017 | #212 | Pigott 2017 |
| 1. **Uganda Tourism Sector COVID-19 Response, Recovery and Sustainability Strategies: Lessons from Previous Virus Disease Outbreaks** | Francis, M.; Jim, A.; Joseph, O. | 2021 | #907 | Francis 2021 |
| 1. **Effects of environmental change on zoonotic disease risk: an ecological primer.** | Estrada-Peña A; Ostfeld RS; Peterson AT; Poulin R; de la Fuente J | 2014 | #2788 | Estrada-Peña 2014 |
| 1. **A Coupled Human and Natural Systems Framework to Characterize Emerging Infectious Diseases-The Case of Fibropapillomatosis in Marine Turtles.** | Manes C; Carthy RR; Hull V | 2023 | #1529 | Manes 2023 |
| 1. **Preparedness for emerging infectious diseases: pathways from anticipation to action.** | Brookes VJ; Hernández-Jover M; Black PF; Ward MP | 2015 | #2720 | Brookes 2015 |
| 1. **Global and regional governance of One Health and implications for global health security.** | Elnaiem A; Mohamed-Ahmed O; Zumla A; Mecaskey J; Charron N; Abakar MF; Raji T; Bahalim A; Manikam L; Risk O; Okereke E; Squires N; Nkengasong J; Rüegg SR; Abdel Hamid MM; Osman AY; Kapata N; Alders R; Heymann DL; Kock R; Dar O | 2023 | #1596 | Elnaiem 2023 |
| 1. **A One Health-based Conceptual Framework for comprehensive and coordinated prevention and preparedness to health threats** | Dente, M.G.; Riccardo, F.; Milano, A.; Robbiati, C.; Agrimi, U.; Morabito, S.; Carere, M.; Marcheggiani, S.; Mantovani, A.; Mancini, L.; Villa, L.; Monaco, M.; Scavia, G.; Cubadda, F.; Declich, S. | 2022 | #3994 | Dente 2022 |
| 1. **The role of ecosystems in mitigation and management of COVID-19 and other zoonoses.** | Everard M; Johnston P; Santillo D; Staddon C | 2020 | #2117 | Everard 2020 |
| 1. **Advances and Limitations of Disease Biogeography Using Ecological Niche Modeling.** | Escobar LE; Craft ME | 2016 | #2561 | Escobar 2016 |
| 1. **Grappling with (re)-emerging infectious zoonoses: Risk assessment, mitigation framework, and future directions** | Gwenzi, W.; Skirmuntt, E.C.; Musvuugwa, T.; Teta, C.; Halabowski, D.; Rzymski, P. | 2022 | #6637 | Gwenzi 2022 |
| 1. **Use of meteorological data in biosecurity.** | Hemming D; Macneill K | 2020 | #2069 | Hemming 2020 |
| 1. **Data and tools to integrate climate and environmental information into public health.** | Ceccato P; Ramirez B; Manyangadze T; Gwakisa P; Thomson MC | 2018 | #10911 | Ceccato 2018 |
| 1. **Eco-social processes influencing infectious disease emergence and spread.** | Jones BA; Betson M; Pfeiffer DU | 2017 | #2560 | Jones 2017 |
| 1. **What needs to be done to control the spread of Middle East respiratory syndrome coronavirus?** | Edelstein, M.; Heymann, D.L. | 2015 | #5517 | Edelstein 2015 |
| 1. **Filovirus serosurvey following an outbreak of marburg hemorrhagic fever - Ibanda and Kamwenge Districts, Uganda, 2007** | Farnon, E.C.; Adjemian, J.A.; Kansiime, E.; Rahman, J.E.; Bwire, G.S.; Kahirita, S.; Kagirita, A.; Wamala, J.F.; Rollin, P.E. | 2009 | #860 | Farnon 2009 |
| 1. **Process Review for Development of Quantitative Risk Analyses for Transboundary Animal Disease to Pathogen-Free Territories.** | Miller J; Burton K; Fund J; Self A | 2017 | #2454 | Miller 2017 |
| 1. **Vulnerability assessment tools for infectious threats and antimicrobial resistance: a scoping review protocol.** | Jeleff M; Lehner L; Giles-Vernick T; Dückers MLA; Napier AD; Jirovsky E; Kutalek R | 2019 | #2196 | Jeleff 2019 |
| 1. **GIS and Remote Sensing for Malaria Risk Mapping, Ethiopia** | Ahmed, A | 2014 | #14115 | Ahmed 2014 |
| 1. **Reemergence of Marburgvirus disease: Update on current control and prevention measures and review of the literature.** | Elsheikh R; Makram AM; Selim H; Nguyen D; Le TTT; Tran VP; Elaziz Khader SA; Huy NT | 2023 | #3 | Elsheikh 2023 |
| 1. **Decision-support tools to build climate resilience against emerging infectious diseases in Europe and beyond** | Rocklöv, J.; Semenza, J. C.; Dasgupta, S.; Robinson, E. J. Z.; Abd El Wahed, A.; Alcayna, T.; Arnés-Sanz, C.; Bailey, M.; Bärnighausen, T.; Bartumeus, F.; Borrell, C.; Bouwer, L. M.; Bretonnière, P. A.; Bunker, A.; Chavardes, C.; van Daalen, K. R.; Encarnação, J.; González-Reviriego, N.; Guo, J.; Johnson, K.; Koopmans, M. P. G.; Máñez Costa, M.; Michaelakis, A.; Montalvo, T.; Omazic, A.; Palmer, J. R. B.; Preet, R.; Romanello, M.; Shafiul Alam, M.; Sikkema, R. S.; Terrado, M.; Treskova, M.; Urquiza, D.; Lowe, R. | 2023 | #10734 | Rocklöv 2023 |
| 1. **Advancing One human-animal-environment Health for global health security: what does the evidence say?** | Zinsstag J; Kaiser-Grolimund A; Heitz-Tokpa K; Sreedharan R; Lubroth J; Caya F; Stone M; Brown H; Bonfoh B; Dobell E; Morgan D; Homaira N; Kock R; Hattendorf J; Crump L; Mauti S; Del Rio Vilas V; Saikat S; Zumla A; Heymann D; Dar O; de la Rocque S | 2023 | #1598 | Zinsstag 2023 |
| 1. **Tracking the distribution and impacts of diseases with biological records and distribution modelling** | Purse, B.V.; Golding, N. | 2015 | #7150 | Purse 2015 |
| 1. **Mapping Potential Amplification and Transmission Hotspots for MERS-CoV, Kenya.** | Gikonyo S; Kimani T; Matere J; Kimutai J; Kiambi SG; Bitek AO; Juma Ngeiywa KJZ; Makonnen YJ; Tripodi A; Morzaria S; Lubroth J; Rugalema G; Fasina FO | 2018 | #198 | Gikonyo 2018 |

Supplemental Table 4 (ST4): Environmental drivers and pathogen transmission routes

| Environmental Driver | Pathogen | Transmission Route | Linkage to climate change | Examples of cited evidence (epidemiological, ecological, and/or serological) | Literature Reference Source examples |
| --- | --- | --- | --- | --- | --- |
| 1. Climate Change^3,4,24,33–55^ | Crimean-Congo haemorrhagic fever virus (CCHFV), Middle East respiratory syndrome coronavirus (MERS-CoV), Marburg virus (MARV) | - Increase in feeding frequency of vectors like CCHFV-infected ticks due to rising temperatures - Population migration and herd mixing near wildlife watering points increases potential of spillover events from MERS-CoV reservoir hosts (dromedary camels) to humans and/or among livestock. - Changes in wind pattern facilitates condition for pathogen dispersal across geographic boundaries - Ecological imbalance caused by climate change results in mixture of different specie/viral strains and cross-species disease transmission.^41^ | n/a | - Dry season reported as a major risk factor for exposure to CCHFV in Kenya (OR: 7.197; 95% CI: 0.813 – 63.65).^37^ - Highest CCHFV seropositivity reported in camels in central Sudan residing in a locality characterized by dry climate and temperature variations.^4^ - Climate change, and climate-induced drought and desertification reported as risk factors for tick distribution in Sudan.^38^ - Climate change and land use change (due to climate-related expansion of arid regions and increase in pastoralism) were found to be spatially correlated with MERS-CoV cases in Kenya from 2016-2018.^43^ - Findings from ecological niche modeling (ENM) studies report influence of climate change on geological distribution of MARV (correlated with seasonal temperature variations).^36,39,40^ | - Lawrence et al. 2024 - Elsheikh et al. 2023 - Blanco-Penedo et al. 2021 - Shuaib et al. 2020 - Suliman et al. 2017 - Peterson et al. 2016 - Kimaro et al. 2013 - Peterson et al. 2006 |
| 1. Migration Patterns (Human, animals, wildlife).^4,6,20,25,28,37,41,48,49,51,56–78^ | MERS-CoV, CCHFV, MARV | - Migratory birds in search of new favorable habitat transport Crimean-Congo haemorrhagic fever (CCHF)-infected ticks to non-endemic areas - MARV-infected Fruit bats migration from natural reservoirs to new roosting and foraging sites increases spillover risk upon human and livestock contact with infected bats drooping - Climate change induced migration of MERS-CoV infected dromedary camel and other livestock to communal watering points/grazing land facilitates cross-species pathogen transmission - Transboundary movement of people and livestock along trade routes | Impacted by climate change with an increase in human displacement and non-human species movement | - Findings from knowledge, attitudes, and practices (KAP) survey in Garissa and Isiolo counties, Kenya on migration of camels to communal watering points, grazing areas and marketing points as high risk driver of the spread of MERS-CoV.^57^ - Findings from a seroprevalence study in cattle (31% seropositivity): Population and livestock migration patterns from arid to semi-arid regions of Kenya to riverine areas facilitate cross-species transfer of pathogens from one region to another.^37^ - Livestock movement associated with CCHFV seroprevalence in Madagascar.^6^ - Mass migration of livestock across Kenya in search of grazing land increases potential for dispersal of tick-borne CCHFV.^72^ - Highest CCHF prevalence reported in Tana River county associated with formation of flood plains during floods and increased temperature favorable to disease transmission; viral spread is further exacerbated by convergence of different species for water and pasture during dry season.^60^ - Livestock movement associated with high CCHFV exposure risk in Uganda and Kenya;^56,58^ and MERS-CoV exposure risk in Ethiopia^59^ and across East Africa.^64,67^ - Increase in MERS seropositivity from Western Kenya to Northeastern and eastern regions associated with increase in cross-border nomadic dromedary camel population.^61^ - Recent study reported the. First evidence of CCHFV seropositivity among rodents (in addition to livestock and humans) in Kenya [6.9%: (6/93)].^20^ | - Omoga et al. 2023 - Lule et al. 2022 - Othieno et al. 2022 - Chiuya et al. 2021 - Blanco-Penedo et al. 2021 - Roess et al. 2015 - Tigoi et al. 2015 - Corman et al. 2014 - Andriamandiby et al. 2011 |
| 1. Habitat Encroachment^11–15,36,42,47,72,78–119^ | MARV, MERS-CoV | - Pathogen spillover from reservoirs hosts (Fruit bats) roosting along the periphery of caves and mines to humans in contact with fruit bats and/or bat secretions | Contributes to | - Significant association between previous mining activity and prior MARV infection (OR: 25.1; 95% CI 5.4 – 118).^91^ - Miners and family members have 2.2 times the risk of filovirus infection (RR: 2.2; 95 CI 0.98 – 4.7).^90^ - Visits to mines and caves inhabited by fruit bats (RR:2.5; 95% CI: 1.12 – 5.66).^90^ - Miners in Western Uganda have 5.4 times the risk of filovirus seropositivity compared to non-miners in Central Uganda (RR: 5.4; 95% CI: 1.5 – 19.7).^89^ - Association between mining and filovirus seropositivity (AOR: 3.4; 95% CI: 1.3 – 8.5); and entering mines and filovirus seropositivity (AOR: 3.1; 95% ci: 1.2 – 8.2).^89^ - Association between Marburg virus disease (MVD) seropositivity and contact with cave roosting bats (OR: 1.2; 95% CI: 1.1 – 1.5).^87,88^ - Linkage of frequent salt mining and manure collection activities by livestock keepers, during visits to caves inhabited by Rousettus spp. Bats, as a primary risk factor for spillover of MARV to humans.^12,92^ | - Rugarabamu et al. 2022 - Rugarabamu et al. 2021 - Nyakarahuka et al. 2020 - Nyakarahuka et al. 2017 - Farnon et al. 2009) - Adjemian et al. 2011 - Sang & Dunster, 2001 |
| 1. Observed Ecological changes due to agricultural intensification^30,34,43,52,56,118,120–123^ | CCHFV, MERS-CoV, MARV | Transmission from *Hyalommma marginatum* and *Rhipicephalus appendiculatus* tick species | Contributes to [associated with increases in greenhouse gas (GHG) emissions) for e.g., poultry production requires use of fossil fuels, and manure use and storage results in emission of GHGs. (Drozdz et al., 2020) and impacted by (increase in extreme weather events results in intensified agricultural practices to improve crop yields). | - Based on eco-epidemiological study, significant predictor of tick transmission in Uganda reported to be agricultural land use, vegetation seasonality (NDVI mean and seasonality), and precipitation and temperature factors; high environmental suitability for CCHFV-infected *R. appendiculatus* tick.^56^ - CCHFV seroprevalence of 10.3% (7-8-13.3) in humans; 69·7% (65·1–73·4) in cattle^56^ - Significant association between CCHFV seroprevalence and proximity to agricultural land; compared to anthropogenic variables, inclusion of environmental variables like enhanced vegetation index, land surface temperature and percent sand-soil composition were better predictors of CCHFV seroprevalence among livestock (31.4%).^121^ - 90% MERS-CoV seroprevalence among camel population in Kenya (100% in Marsabit county); Ecological changes due to intensive agricultural practices increases risk of MERS-CoV transmission to humans.^30^ | - Telford et al. 2023 - Lule et al. 2022 - Magenas et al. (2022) - Munyua et al. 2017 |
| 1. Deforestation and Reforestation^36,72,124^ | MARV | Impacts of deforestation due to mining and logging activities results in the redistribution of bats habitat and facilitate MVD emergence and spread to non-endemic areas | Contributes to climate change | - ENM and species distribution modeling (SDM studies) reported a significant correlation between biological reservoirs including filoviruses and deforestation (AUC: 0.978).^124^ | - Jagadesh et al. 2020 |
| 1. Animal Husbandry practices^1,5,18,19,21,29,47,49,61,70,71,74,125–133^ | CCHFV, MERS-CoV | Herd mixing of livestock with disease infected wildlife; increased interaction of immunologically naïve and infected animals during transportation of livestock from different herds and in holding pens^125^ | Contributes to | - Higher MERS-CoV seroprevalence in camels raised on pastoral production system (69%; 95% CI: 66.5 - 71.6) compared to ranching production system (10.1%; 95% CI: 4.9 – 15.3).^74,126^ - Cattle raised on open grazing system reported to be 27 times more at risk of CCHFV infection compared to cattle raised in closed grazing system (OR: 27.22; 95% CI: 7.46- 99.24). ^5^ - Association between poor animal husbandry of famers in contact with donkeys and CCHFV exposure (OR: 1.92; 95% CI: 1.05 - 3.72).^21^ - 95% CCHFV seroprevalence rate reported in African buffalo raised in closed wildlife grazing systems in Kenya compared to seropositivity rates of 29% and 46% reported for closed integrated and open integrated grazing systems respectively.^19^ | - Ogoti et al. 2024 - Obanda et al. 2021 - Sitawa et al. 2020 - Gardner et al. 2019 - Ibrahim et al. 2015 - Lwande et al. 2012 |
| 1. Livestock Overgrazing^2,134,135^ | CCHFV, MARV | Expansion of livestock grazing lands to overlap into areas typically inhabited by wildlife provides more opportunity for cross-species pathogen transmission | Contributes to | - People living close to livestock grazing fields reported as 18 times more likely to be CCHF case patients than controls (OR: 18, 95% CI: 3.2 - ∞) - Cross sectional study and focus group discussion focused on community perspective reported lowland grazing, settling in caves during we season, and salt mining and utilization of bat droppings as livestock fertilizer as major risk factors for MVD emergence in Uganda^135^ | - Mirembe et al. 2021 - Siya et al. 2019 |
| 1. Biodiversity loss^42,49,104,136^ | CCHFV | - Expansion of livestock grazing land due to climate change results in biodiversity loss including loss of species serving basic ecosystem function (for e.g. disease control). - land use changed linked to increase in zoonotic reservoir host species and a decline in non-reservoir species/wildlife population - Declines in vulture population around abattoirs increase opportunities for pathogen transmission from infected ticks feeding on livestock in abattoirs | Impacted by | - Reported declines in vulture population linked to rise in diseases spread via abattoirs in Eastern Africa including tick-borne diseases.^136^ - Findings from micro-global positioning study support earlier evidence about decrease in non-reservoir species due to land use change | - Rodarte et al. 2023 - Amman et al. 2023 |
| 1. Irrigation Practices^72^ | CCHFV, MERS-CoV | Contained water sources (independent of seasonal rain fall patterns) prolongs pathogen vector breeding periods and provides more vector breeding habitat that result in increased risk of disease transmission | Impacted by, and contributes | Human settlements around irrigation sites linked to increased risk of CCHFV transmission in Kenya.^72^ | Sang & Dunster 2001 |
| 1. Human-mediated transport of pathogens across geographic boundaries (“pathogen pollution”)^93,125^ | MARV, MERS-CoV | Transport of MERS-CoV infected nomadic camels to abattoir hubs increases MERS-CoV exposure risk to abattoir workers | Impacted by, and contributes to | Seasonal factors (such as dry seasom) were associated with increased MERS-CoV RNA positivity in camels [36/2711 (1.3%] and biphasic incidence of MER-CoV infections from nomadic camels to abattoir workers in Northern Kenya [October 2022 (7/60, 11.7%); and February 2023 (7/58, 12.1%)].^125^ | Ogoti et al. 2024 |

Supplemental Note 1 (SN 1): Summary of Risk Frameworks and Areas of Application

1. Risk Mapping-Based Assessment Tools: Reported application of conventional risk maps include for use as a forecasting system and decision support tool (DST) during the 2006-2007 Rift Valley fever (RVF) outbreaks linked to El-Nino episodes in Kenya;^137^ identifying and mapping MERS-CoV transmission hotspots and risk nodes along the camel value chain in Kenya;^63^ and anthrax risk mapping to inform risk communication and decision making.^138^ Specialized risk mapping approaches like ecological niche modeling (ENM) are reportedly used for filling surveillance data gaps and other gaps in conventional risk mapping approaches. Some studies reported on the application of ENM combined with remote sensing technologies to enhance environmental monitoring data including for flood and other environmental hazard detection in the East Africa region.^139,140^ Related to ENM application for environmental monitoring, a study carried out in Uganda found that socio-environmental variables like land cover, average annual change in nighttime light index, EVI, Normalized Difference Vegetation Index (NDVI) and percent sand-soil content have the best predictive accuracy for risk of CCHFV transmission among livestock;^121^ and one study described its application for determining environmental and socio-ecological suitability for CCHF infections in Uganda.^56^ Another study on the environmental suitability for risk of MERS-CoV transmission found the most significant predictor variables to be bare land coverage, forest coverage, and population density;^141^ and a review article highlighted the geological distribution of MARV and its association with climate change (in relation to pathogen distribution across precipitation areas on the African continent) with a reported correlation between MVD and seasonal temperature variations characterized by low to moderate temperature and precipitation.^36,107^ ENM also associates temperature seasonality, rainfall, and vegetation indices as key determinants of the spatial distribution of MARV.^39,107,115,142,143^
2. *Multivariable risk assessment and management frameworks*: Included in the review findings are risk assessment and risk management frameworks that incorporate a combination of different categories of variables for multihazard risk assessment. These frameworks include the Index For Risk Management Epidemic Risk Index (INFORM ERI), an open-source risk assessment tool adopted from the Index for Risk Management Global Risk Index (INFORM GRI) and used to address gaps in research on risks of climate-related hazards and exposures including epidemic risks of four viral hemorrhagic fevers of epidemic potential;^144,145^ Spatial Multicriteria Decision Analysis tool utilized for assessing emergence of West Nile Virus in China;^146^  WHO Health Emergency and Disaster Risk Management (EDRM) framework that utilizes an all-hazards risk management approach to mitigating the health risks and consequences of emergencies including climate-induced infectious disease outbreaks;^147^ UNDRR Technical guidance framework on application of climate information for comprehensive risk management;^148^ and integrated risk and vulnerability assessment framework developed for assessing the risk of climate change associated malaria transmission at the community level in East African highlands.^149^
3. *Qualitative and semi-quantitative risk tools based on expert opinions*: Some studies reported the use of expert-informed qualitative multisectoral risk assessment tools such as the Tripartite Joint Risk Assessment Operational Tool ( JRA OT) used for addressing zoonotic biological threats at the animal-human-environment interface across different countries including for the risk assessment of rabies and influenza in Jordan,^150^ and as part of the OH zoonotic disease prioritization exercise in Ukraine.^151^ Reported limitations of the tool include real-time cross-sectoral data gaps due to gaps in surveillance systems across the country thus contributing to a higher level of uncertainty associated with the Joint Risk Assessment (JRA) process and the need to strengthen the environment-interface by better integrating the environment sector into the JRA process.^150^

Other risk assessment tools informed by expert opinions include hybrid risk ranking tools such as the Spillover Viral Risk Ranking tool used to assess the spillover and pandemic potential of zoonotic viruses,^152^ to track potential reservoirs of emerging pathogens^153^ and to assist countries with priority zoonotic diseases risk ranking efforts to inform resource allocation;^154^ infectious disease seeker software tool used for predicting, assessing, and comparing different outbreaks;^139,155^ a risk classification methodological tool used for classifying influenza viruses;^156^ semi-quantitative risk assessment tool utilized for the risk assessment of spillover routes and disease amplification;^157^ and the Africa CDC Risk Classification and Prioritization of Epidemic-Prone Diseases tool to identify priority diseases of epidemic potential to inform resource allocation and implementation of risk mitigation measures.^158^

1. *Quantitative-based environmental risk assessment tools:* Application of environmental risk assessment tools utilized for detecting zoonotic biological threats include environmental vulnerability and preparedness risk assessment checklist;^159^ Quantitative Microbial Risk Assessment (QMRA) to investigate potential health risks of severe acute respiratory syndrome coronavirus 2 (SARS-CoV-2) for vulnerable groups such as wastewater treatment plant workers and for scenario planning.^160^ Another study reported on the application of QMRA in combination with remote sensing technology to monitor cross-species population movement and potential for disease transmission,^161^ and for the assessment of pathogens in aquatic environments.^162^ Using a similar environmental approach, Bayesian Belief Networks were used to make disease risk estimation, characterization, and prediction for climate adaptation.^163^

Supplemental Table 5 (ST5): Summary of risk frameworks/methodological approaches and areas of application

| Integrated  Risk Framework/  Methodological  Approach | Example Description | Unique Features and Benefits | Example areas of application in the region | Extent of Geographic Coverage | Highlighted Gap areas |
| --- | --- | --- | --- | --- | --- |
| Ecological Niche Modeling (ENM)/ Species Distribution Modeling (SDM)^36,39,107,115,118,124,139,141,143,145,153,164–176^ | Based on environmental interpolations/estimations of potential distributions; useful for identifying at-risk areas by utilizing data on environmental correlation found in areas with strong disease surveillance and reporting coverage to make risk predictions in areas with limited surveillance and reporting and thus little knowledge about disease transmission potential.^166^ | - Adopts different types of Algorithms including Maximum Entropy (MaxEnt)^107,141,175^, Boosted Regression Trees^56,115^, Random Forest, Genetic Algorithm for Rule-set Prediction (GARP) - Uses both presence only occurrence data and absence data to provide an estimation of both potential distribution (ENM) and actual distribution (SDM) of a species/pathogen - Predicts potential hotpots and suitable environments for future outbreaks helpful for prioritizing resource-limited high-risk areas for surveillance, prevention, preparedness, and control efforts. | - For detection of potential risk of MARV spillover in countries across SSA.^115^ - Potential for human rabies transmission in non-endemic area.^167^ - Potential outbreaks of filovirus Uganda.^90^ - Potential tick host of Crimean-Congo haemorrhagic fever virus (CCHFV) and widespread exposure to the virus in Uganda.^56^ - Risk of Middle East respiratory syndrome coronavirus (MERS-CoV) transmission globally.^141^ | Global, National, Regional, Local (sub-national) | Need to improve ENM validation systems, and account for gaps in reporting of occurrence data across countries to reduce bias in distribution models |
| Geospatial Analysis and Remote Sensing data systems;^137,139,140,142,161,163,177–183^ and Spatial Multicriteria decision analysis tool (SpatMCDA)^138,146,174,184,185^ | Useful for large-scale environmental mapping and surveillance including for the identification of risk factors contributing to multiple clusters of emerging and/or reemerging infectious diseases (EIDs) such as coronavirus emergence.^182^ | - EPIDEMIA provides an interdisciplinary approach to malaria surveillance, forecasting and decision support to inform malaria prevention, control and elimination in Ethiopia.^178^ - SpatMCDA is utilized for assessing areas at risk of EIDs such as West Nile virus especially in resource limited settings.^146^ - Used to determine the spatial relationship of EIDs to variables such as climate, agriculture and sociodemographic factors.^43^ | Amhara region, Ethiopia  Northwestern, Eastern, and Southern China  MERS-CoV seroprevalence in Kenya | National and Sub-national | Need for the development of a comprehensive, open access database for sharing, storing, and accessing remotely-and proximally sensed data sources |
| Traditional Risk Mapping^137,186–188^ | Use of 2-3 dimensional visualizations depicting high vs. no risk areas^138^; Based on disease occurrence density and spatial interpolations | Used as a disease forecasting system to make predictions about areas with risk of disease emergence to inform risk communication efforts | Food and Agriculture Organization (FAO) Rift Valley fever (RVF) Decision support tools for enhancing capacity for early warning and forecasting of RVF in high-risk countries (such as Kenya, Tanzania, Uganda) in the East Africa region.^137,188^ | National, Sub-national | Does not account for risk in areas with limited/lack of data on disease occurrence (absence data) due to low surveillance coverage |
| Integrated Risk and Vulnerability Assessment^149^ | Conceptual framework utilizing a combination of variables to assess the interplay among biophysical  (especially climate change) and socio-economic and cultural factors on malaria transmission in East Africa | Adopts the use of a combination of variables to assess risk of vector-borne disease transmission at the community level | Community level risk of malaria transmission due to climate change in the East Africa region.^149^ | Community level | The need for the adoption of a combination of empirical quantitative and qualitative data sources including from communities, and expert opinion, to ensure the development of a more robust risk analysis model for decision making |
| Joint Risk Assessment Operational Tool (JRA OT)^150,151,189^; other multisectoral risk assessment approaches^190–192^ | Useful for collating cross-sectoral expert opinion to characterize disease risk using a mostly qualitative data | Helpful guide for bringing together all relevant sectors to assess shared threats and to inform risk management and risk communication; and resource allocation^193^ | Joint risk assessment (JRA) of rabies and Avian influenza in Jordan.^150^ | National and Sub-national | - Need to expand the scope of the tool to threats related to environmental hazard and exposures including better utilization of the tool for assessing the impact of environmental hazards on the spillover, emergence, and spread of zoonotic biological threats; better integration of the environmental sector as a key technical partner in the operationalization of the tool; integrating the United Nations Environment Programme (UNEP) and national environmental sector risk assessment processes to the JRA OT operationalization process. - Reported gaps in surveillance systems increase uncertainty level associated with the JRA process - Addressing real-time/near real-time data gaps to better quantify risk level |
| Index for Risk Management Global Risk Index (INFORM GRI/ Epidemic Risk Index (INFORM ERI)^144,194^ | Used to address gaps on risks of climate-related hazards and exposures in relation to epidemic risks to inform decision-making.^194^ | Adapted from the Index for Risk Management Global Risk Index (INFORM GRI) to specifically analyze environmentally driven biological threat exposure risks, population vulnerabilities, and lack of coping capacities to inform the implementation of appropriate risk mitigation and risk communication strategies | Application by Pigot et al. (2017) show how an index case escalated into a widespread epidemic and areas of potential susceptibility at local, regional, and global level.^195^ | Global, Regional, National | Need to expand application to diverse range of pathogens with epidemic and pandemic potential |
| Risk Ranking Tools^153–158,196^ | - SpillOver Viral Risk Ranking Framework: Web-based risk assessment tool used to evaluate and assess the zoonotic spillover and pandemic potential of novel viruses.^196^ - Infectious Disease seeker software tools: used for predicting, assessing, and comparing different outbreaks.^155^ - Risk Ranking and Prioritization of Epidemic-Prone Diseases tool: used for prioritization of diseases of epidemic potential to inform resource allocation and implementation of risk mitigation interventions.^158^ | - Open-source and adaptable platform for collating data on spillover risk factors and assigns a comparative risk score for viruses of wildlife origins | - Can be used in combination with ENM to better characterize emerging pathogen reservoir host species | Global, Regional | - Lack of/limited data on wildlife source of animal-human spillover transmission risk reduces the accuracy of risk ranking tools (resulting in over- or under-estimation of assigned risk score); inconsistencies with reporting on pathogen host species - Data gaps serve as a hindrance to properly characterizing risk factors |
| Other environmentally focused risk assessment methodological tools [Quantitative Microbial Risk Assessment (QMRA),^157,160–162^ Bayesian Networks Modeling]^124,157,163^ | Utilizes a combination of quantitative and qualitative data for environmental monitoring | Combines qualitative expert knowledge with quantitative data including remote sensing data for model development | - Adoption of QMRA to investigate health risks of severe acute respiratory syndrome coronavirus 2 (SARS-CoV-2) to vulnerable groups such as wastewater treatment plant workers.^160^ - Use of Bayesian Networks for risk assessment of spillover routes, disease amplification and spread in South Korea.^157^ | National | - Data intensive: reported challenges with data gaps in both developed and developing countries - Need to better account for the perspective of stakeholders in model design, implementation, and communication of findings |

*Supplemental Note 2 (SN2): Implications for public health practice*

Based on the integrative review findings, our proposed integrated risk analysis (IRA) framework primarily incorporates a combination of the ecological, meteorological, biological, and socio-anthropogenic drivers that increase pathogen spillover risk and disease emergence/reemergence into the risk assessment and risk characterization steps. In line with the WHO Health EDRM framework,^197^ the IRA framework also addresses the need for proactive health systems resilience building as a risk mitigation strategy to mitigate the impact of environmentally driven biological events of potentially catastrophic magnitude. This new framework also accounts for recommendations for an integrated OH-based conceptual framework informed by expert opinions to facilitate operationalization of OH. Thus, the framework addresses the need to enhance the integration of the environment sector into the OH approach for the implementation of pandemic prevention and preparedness activities including climate-resilient health systems resilience building measures.^190,198–200^

The figure below *(Figure 1*) describes the application of the newly conceptualized integrated risk analysis (IRA) to provide a coordinated cross-sectoral response to a hypothetical cluster of future cases of Crimean-Congo haemorrhagic fever (CCHF) infections in Kenya. The scenario presented is in line with the integrative review findings on past CCHF outbreaks across countries in the East Africa region. The IRA framework incorporates key findings from the review about addressing gaps in existing risk analysis framework and methodological tools to address environmentally driven zoonotic biological threats at the animal-human-environment interface using an integrated approach to risk analysis.

Figure 1: Application of the newly conceptualized Integrated Risk Analysis framework model


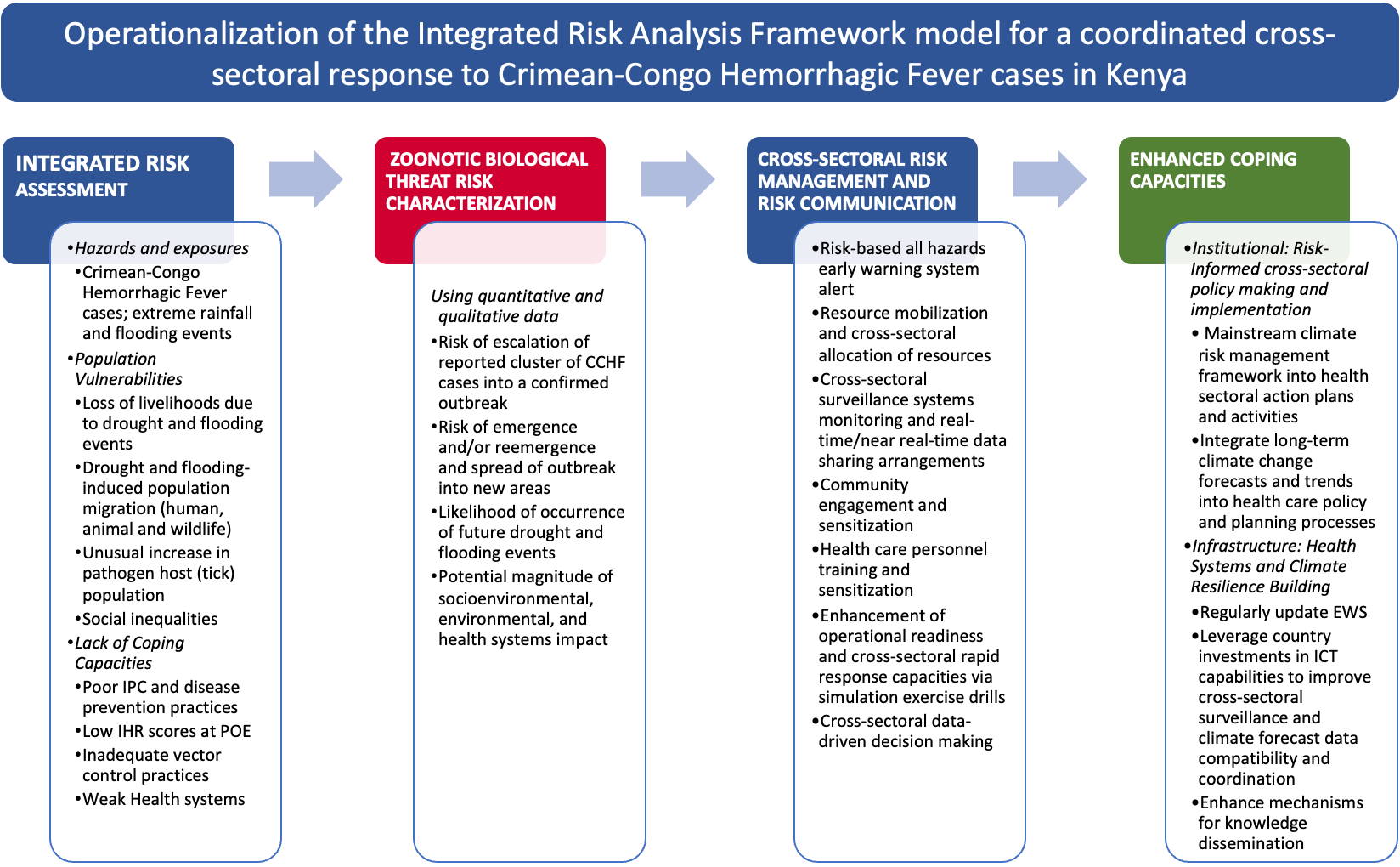


**Box 1 - A Case Study: Operationalization of a newly conceptualized Integrated Risk Analysis (IRA) framework for a coordinated cross-sectoral response to the reemergence of Crimean-Congo Haemorrhagic Fever (CCHF) in two high-risk counties in Kenya**

Converging risk drivers

*Hazards and exposures*

The Ministry of Health (MoH) recently received an alert about suspected cases of CCHF in two counties in Kenya. As an integral part of the cross-sectoral response to the reported cluster of cases, the Ministry of Environment, Climate Change and Forestry closely monitors national climate data and shares information on common climate-related hazards (flooding and drought) events at the national and county levels that are known to have an impact on vector-borne disease transmission patterns. The risk analysis also considers past trends and future projections about climate change and climate-related multihazard occurrence, based on ecological niche modeling (ENM)/species distribution modeling (SDM) data, climate models, and geospatial and remotely sensed data. This information is included in the analysis of epidemiological surveillance data from the MoH and the Ministry of Agriculture and Livestock Development (MALD) on suspected and confirmed cases of CCHF in animals and humans.

*Population vulnerabilities*

In both affected counties and cross-border counties with no known reported case of CCHF, receding lake levels, drying of the rivers and other wetlands puts local communities that rely on fishing and agriculture as a primary source of income in a more vulnerable state. With the approaching drought season, there has been mass population migration from the arid and semi-arid zones of the country towards the highlands to escape the socioeconomic impact of droughts due to agriculture and livestock loss. Similarly, past incidences of flooding events due to extreme rainfalls are causing frequent land degradation, soil erosion and water logging of crops which result in reduced crop yields, increased food insecurity, and an unusual increase in tick population. In addition to community displacement, the effects of these climate-related hazards provide an economic incentive for mass human, animal, and wildlife population migration to other areas in search of new habitat. Hence, available data on changing migration patterns, economic development, and social inequality will be accounted for in the risk assessment.

*Lack of coping capacities*

At the devolved county government levels, poor infection, prevention and control (IPC) and disease prevention practices, and low levels of International Health Regulations (IHR) core capacities scores at points of entry have been identified as key weaknesses in coping capacities. In addition, underlying causes of vulnerabilities include gaps in water and sanitation services, weaknesses in health systems capacity to address climate-related hazards, poorly implemented veterinary regulations, as reported by the MALD, and inadequate vector control practices.

Risk characterization

Based on the initial assessment of available cross-sectoral data, the risk characterization accounts for the level of CCHF exposure, population vulnerabilities, and capacities at national and county level. Exposure levels consider the risk of escalation of reported clusters of cases into a confirmed outbreak, and the emergence/reemergence of the outbreak into new areas. Additionally, the likelihood of occurrence of future climate-related hazards (droughts and floods) and the potential magnitude of impact in terms of socioeconomic, environmental, and health systems impact is also included in the risk characterization.

**Box 1 (continued) - A Case Study: Operationalization of a newly conceptualized IRA framework for a coordinated cross-sectoral response to the reemergence of CCHF in two high-risk counties in Kenya**

Cross-sectoral Risk Management and Risk Communication

Following a heightened situational awareness about the reported clusters of CCHF cases, an early warning system (EWS) is triggered to indicate the need to take concrete actions (specific to the local context) including targeted mobilization and allocation of resources across different line ministries to enable further investigations, close monitoring of surveillance systems and cross-sectoral sharing of data in real time/near-real time to further update the risk assessment as more information becomes available. In addition, community engagement, sensitization and surveillance of unusual events is prioritized to alert health authorities and to ensure inclusion of the most-affected populations in the risk management decision-making process. As part of current risk mitigation and future risk reduction efforts, resources are also mobilized for improving health systems preparedness capacity including for the sensitization and training of health care personnel on improving IPC practices, while ensuring safe treatment of identified cases, and for the monitoring and prevention of climate-related diseases. Plans are also put in place to enhance operational readiness and rapid response capacity via simulation drills in preparation to respond to large-scale outbreaks and other climate-related hazards. This involves the use of ENM/SDM, climate modeling, remotely sensed data, and surveillance data on environmental drivers for data-driven decision making and awareness raising to ensure increased political commitment and sustained investments towards prevention of climate-related hazards and future preparedness planning using a OH approach.

Outlook: Enhanced Coping Capacities

The actions to be taken to enhance capacities address underlying risk drivers of CCHF emergence/reemergence and spread in affected counties, while focusing on future preparedness, mitigation, and long-term resilience building efforts. Proactive actions for strengthening coping capacities include regularly updating EWS based on most recent data on environmental drivers of EIDs to trigger a faster response to future outbreaks; improving the political, legal, and regulatory environment to ensure the adoption of effective OH policies (including climate-smart agriculture policies and integration of long-term climate change forecasts and trends into health care policy and planning processes); and implementation of OH risk-based activities under existing policies such as the National Action Plan for Health Security (NAPHS) and country environment action plan. Such activities include integrating cross-sectoral surveillance data and enabling better use of environmental, climate change, public health, and animal health data at the national and county level, mainstreaming climate risk management into health sectoral plans and activities, in line with Kenya’s Integrated Climate Risk Management Framework, leveraging on the country’s investments in Information Communication and Technology (ICT), meeting commitments towards improving cross-sectoral knowledge sharing platforms and information management systems to ensure data compatibility and coordination across sectors, and improving mechanisms for community engagement and knowledge dissemination to inform future EIDs risk analysis.

References

1. Nyakarahuka L, Whitmer S, Kyondo J, et al. Crimean-Congo Hemorrhagic Fever Outbreak in Refugee Settlement during COVID-19 Pandemic, Uganda, April 2021. *Emerg Infect Dis*. 2022;28(11):2326-2329. doi:10.3201/eid2811.220365

2. Mirembe BB, Musewa A, Kadobera D, et al. Sporadic outbreaks of Crimean-Congo haemorrhagic fever in Uganda, July 2018-January 2019. *PLoS Negl Trop Dis*. 2021;15(3):e0009213-. doi:10.1371/journal.pntd.0009213

3. Balinandi S, Whitmer S, Mulei S, et al. Clinical and Molecular Epidemiology of Crimean-Congo Hemorrhagic Fever in Humans in Uganda, 2013-2019. *Am J Trop Med Hyg*. 2021;106(1):88-98. doi:10.4269/ajtmh.21-0685

4. Suliman HM, Adam IA, Saeed SI, Abdelaziz SA, Haroun EM, Aradaib IE. Crimean Congo hemorrhagic fever among the one-humped camel (Camelus dromedaries) in Central Sudan. *Virol J*. 2017;14(1):147. doi:10.1186/s12985-017-0816-3

5. Ibrahim AM, Adam IA, Osman BT, Aradaib IE. Epidemiological survey of Crimean Congo hemorrhagic fever virus in cattle in East Darfur State, Sudan. *Ticks Tick Borne Dis*. 2015;6(4):439-444. doi:10.1016/j.ttbdis.2015.03.002

6. Andriamandimby SF, Marianneau P, Rafisandratantsoa JT, et al. Crimean-Congo hemorrhagic fever serosurvey in at-risk professionals, Madagascar, 2008 and 2009. *J Clin Virol*. 2011;52(4):370-372. doi:10.1016/j.jcv.2011.08.008

7. Aradaib IE, Erickson BR, Karsany MS, et al. Multiple Crimean-Congo hemorrhagic fever virus strains are associated with disease outbreaks in Sudan, 2008-2009. *PLoS Negl Trop Dis*. 2011;5(5):e1159-. doi:10.1371/journal.pntd.0001159

8. Dunster L, Dunster M, Ofula V, et al. First documentation of human Crimean-Congo hemorrhagic fever, Kenya. *Emerg Infect Dis*. 2002;8(9):1005-1006. doi:10.3201/eid0809.010510

9. World Health Organization. Marburg virus disease - Rwanda. Disease Outbreak News. 2024. Accessed October 13, 2024. https://www.who.int/emergencies/disease-outbreak-news/item/2024-DON537

10. World Health Organization. Marburg Virus Disease - United Republic of Tanzania. Disease Outbreak News .

11. World Health Organization. Outbreak of Marburg haemorrhagic fever: Uganda, June-August 2007. *Wkly Epidemiol Rec*. 2007;82(43):381-384.

12. Nyakarahuka L, Shoemaker TR, Balinandi S, et al. Marburg virus disease outbreak in Kween District Uganda, 2017: Epidemiological and laboratory findings. *PLoS Negl Trop Dis*. 2018;13(3). doi:10.1371/journal.pntd.0007257

13. Nyakarahuka L, Ojwang J, Tumusiime A, et al. Isolated Case of Marburg Virus Disease, Kampala, Uganda, 2014. *Emerg Infect Dis*. 2017;23(6):1001-1004. doi:10.3201/eid2306.170047

14. Amman BR, Nyakarahuka L, McElroy AK, et al. Marburgvirus resurgence in Kitaka Mine bat population after extermination attempts, Uganda. *Emerg Infect Dis*. 2014;20(10):1761-1764. doi:10.3201/eid2010.140696

15. Albariño CG, Shoemaker T, Khristova ML, et al. Genomic analysis of filoviruses associated with four viral hemorrhagic fever outbreaks in Uganda and the Democratic Republic of the Congo in 2012. *Virology*. 2013;442(2):97-100. doi:10.1016/j.virol.2013.04.014

16. Timen, Aura; Koopmans, Marion; Vossen, Ann; van Doornum, Gerard; Gunther, Stephan; Berkmortel, Franchette, Verduin, Kees; Dittrich, Sabine, Emmerich, Petra; Osterhaus, Albert; van Dissel, Jaap; Coutinho R. Response to Imported Case of Marburg Hemorrhagic Fever, the Netherlands. *Emerg Infect Dis*. 2009;15(8):1171-1175. doi:10.3201/eid1508.090051

17. US Centers for Disease Control and Prevention. History of Marburg virus outbreaks.

18. Hughes EC, De Glanville W, Kibona T, et al. Crimean-Congo Hemorrhagic Fever Virus Seroprevalence in Human and Livestock Populations, Northern Tanzania. *Emerg Infect Dis*. 2024;30(4):836-838. doi:10.3201/eid3004.231204

19. Obanda V, Agwanda B, Blanco-Penedo I, et al. Livestock Presence Influences the Seroprevalence of Crimean Congo Hemorrhagic Fever Virus on Sympatric Wildlife in Kenya. *Vector Borne Zoonotic Dis*. 2021;21(10):809-816. doi:10.1089/vbz.2021.0024

20. Omoga DCA, Tchouassi DP, Venter M, et al. Transmission Dynamics of Crimean–Congo Haemorrhagic Fever Virus (CCHFV): Evidence of Circulation in Humans, Livestock, and Rodents in Diverse Ecologies in Kenya. *Viruses*. 2023;15(9). doi:10.3390/v15091891

21. Lwande OW, Irura Z, Tigoi C, et al. Seroprevalence of Crimean Congo hemorrhagic fever virus in Ijara District, Kenya. *Vector Borne Zoonotic Dis*. 2012;12(9):727-732. doi:10.1089/vbz.2011.0914

22. Munyua PM, Ngere I, Hunsperger E, et al. Low-Level Middle East Respiratory Syndrome Coronavirus among Camel Handlers, Kenya, 2019. *Emerg Infect Dis*. 2021;27(4):1201-1205. doi:10.3201/eid2704.204458

23. Ngere I, Hunsperger EA, Tong S, et al. Outbreak of Middle East Respiratory Syndrome Coronavirus in Camels and Probable Spillover Infection to Humans in Kenya. *Viruses*. 2022;14(8). doi:10.3390/v14081743

24. Ommeh S, Zhang W, Zohaib A, et al. Genetic Evidence of Middle East Respiratory Syndrome Coronavirus (MERS-Cov) and Widespread Seroprevalence among Camels in Kenya. *Virol Sin*. 2018;33(6):484-492. doi:10.1007/s12250-018-0076-4

25. Kandeil A, Gomaa M, Nageh A, et al. Middle East Respiratory Syndrome Coronavirus (MERS-CoV) in Dromedary Camels in Africa and Middle East. *Viruses*. 2019;11(8). doi:10.3390/v11080717

26. Farag E, Sikkema RS, Mohamedani AA, et al. MERS-CoV in Camels but Not Camel Handlers, Sudan, 2015 and 2017. *Emerg Infect Dis*. 2019;25(12):2333-2335. doi:10.3201/eid2512.190882

27. Kiyong’a AN, Cook EAJ, Okba NMA, et al. Middle East Respiratory Syndrome Coronavirus (MERS-CoV) Seropositive Camel Handlers in Kenya. *Viruses*. 2020;12(4). doi:10.3390/v12040396

28. Kiambi S, Walelign E, Nyariki T, et al. Contributions of the MERS-CoV research to One Health operationalization in Ethiopia and Kenya. *J Public Health Afr*. 2022;12(SUPPL 1):16-17. https://www.embase.com/search/results?subaction=viewrecord&id=L638272788&from=export

29. Liljander A, Meyer B, Jores J, et al. MERS-CoV Antibodies in Humans, Africa, 2013-2014. *Emerg Infect Dis*. 2016;22(6):1086-1089. doi:10.3201/eid2206.160064

30. Munyua P, Corman VM, Bitek A, et al. No Serologic Evidence of Middle East Respiratory Syndrome Coronavirus Infection Among Camel Farmers Exposed to Highly Seropositive Camel Herds: A Household Linked Study, Kenya, 2013. *Am J Trop Med Hyg*. 2017;96(6):1318-1324. doi:10.4269/ajtmh.16-0880

31. Munyua PM, Njenga MK, Osoro EM, et al. Successes and challenges of the One Health approach in Kenya over the last decade. *BMC Public Health*. 2019;19(Suppl 3):465. doi:10.1186/s12889-019-6772-7

32. Reusken CB, Messadi L, Feyisa A, et al. Geographic distribution of MERS coronavirus among dromedary camels, Africa. *Emerg Infect Dis*. 2014;20(8):1370-1374. doi:10.3201/eid2008.140590

33. Okesanya OJ, Olatunji GD, Kokori E, et al. Looking Beyond the Lens of Crimean-Congo Hemorrhagic Fever in Africa. *Emerg Infect Dis*. 2024;30(7):1319-1325. doi:10.3201/eid3007.230810

34. Lawrence TJ, Kangogo G, Fredman A, et al. MERS-Coronavirus across Kenya: a spatial examination of social and environmental drivers. doi:10.1101/2023.11.14.23298516

35. Mukhaye E, Akoko JM, Nyamota R, et al. Exposure patterns and the risk factors of Crimean Congo hemorrhagic fever virus amongst humans, livestock and selected wild animals at the human/livestock/wildlife interface in Isiolo County, upper eastern Kenya. *PLoS Negl Trop Dis*. 2024;18(9):e0012083. doi:10.1371/journal.pntd.0012083

36. Elsheikh R, Makram AM, Selim H, et al. Reemergence of Marburgvirus disease: Update on current control and prevention measures and review of the literature. *Rev Med Virol*. Published online 2023:e2461-. doi:10.1002/rmv.2461

37. Blanco-Penedo I, Obanda V, Kingori E, Agwanda B, Ahlm C, Lwande OW. Seroepidemiology of Crimean-Congo Hemorrhagic Fever Virus (CCHFV) in Cattle across Three Livestock Pastoral Regions in Kenya. *Dairy*. 2021;2(3):425-434. doi:10.3390/dairy2030034

38. Shuaib YA, Elhag AMW, Brima YA, et al. Ixodid tick species and two tick-borne pathogens in three areas in the Sudan. *Parasitol Res*. 2020;119(2):385-394. doi:10.1007/s00436-019-06458-9

39. Peterson AT, Samy AM. Geographic potential of disease caused by Ebola and Marburg viruses in Africa. *Acta Trop*. 2016;162:114-124. doi:10.1016/j.actatropica.2016.06.012

40. Peterson AT, Lash RR, Carroll DS, Johnson KM. Geographic potential for outbreaks of Marburg hemorrhagic fever. *Am J Trop Med Hyg*. 2006;75(1):9-15. doi:10.4269/AJTMH.2006.75.1.0750009

41. Kimaro EG, Chibinga OC. Potential impact of climate change on livestock production and health in East Africa: A review. *Livest Res Rural Dev*. 2013;25(7). https://www.scopus.com/inward/record.uri?eid=2-s2.0-84879765125&partnerID=40&md5=a92f8e1fd5f5ed9f89865ce4fe3cd98e

42. Amman BR, Schuh AJ, Akurut G, et al. Micro‒Global Positioning Systems for Identifying Nightly Opportunities for Marburg Virus Spillover to Humans by Egyptian Rousette Bats. *Emerg Infect Dis*. 2023;29(11):2238-2245. doi:10.3201/eid2911.230362

43. Lawrence TJ, Kangogo GK, Fredman A, et al. Spatial examination of social and environmental drivers of Middle East respiratory syndrome coronavirus (MERS-CoV) across Kenya. *Ecohealth*. Published online 2024. doi:10.1007/s10393-024-01684-9

44. Liao H, Lyon CJ, Ying B, Hu T. Climate change, its impact on emerging infectious diseases and new technologies to combat the challenge. *Emerg Microbes Infect*. 2024;13(1). doi:10.1080/22221751.2024.2356143

45. Hughes, EC; Cleaveland, S; Allan, KJ; Kibona, TJ; Lankester, F; Davis, A; de Glanville, WA; Willett B. Crimean-Congo haemorrhagic fever virus seroprevalence in Tanzanian Livestock. In: *Transactions of the Royal Society of Tropical Medicine and Hygiene*. ; 2019.

46. Sargianou M, Papa A. Epidemiological and behavioral factors associated with Crimean-Congo hemorrhagic fever virus infections in humans. *Expert Rev Anti Infect Ther*. 2013;11(9):897-908. doi:10.1586/14787210.2013.827890

47. Mirkena T, Walelign E, Tewolde N, Gari G, Abebe G, Newman S. Camel production systems in Ethiopia: a review of literature with notes on MERS-CoV risk factors. *Pastoralism*. 2018;8(1):30. doi:10.1186/s13570-018-0135-3

48. Ahmed A, Ali Y, Salim B, Dietrich I, Zinsstag J. Epidemics of Crimean-Congo Hemorrhagic Fever (CCHF) in Sudan between 2010 and 2020. *Microorganisms*. 2022;10(5). doi:10.3390/microorganisms10050928

49. Zhu S, Zimmerman D, Deem S. Dromedary camels: Growing zoonotic disease risk at the human-livestock-wildlife interface. *American Journal of Tropical Medicine and Hygiene*. 2018;99(4):406. https://www.embase.com/search/results?subaction=viewrecord&id=L627540743&from=export

50. Deem SL, Fèvre EM, Kinnaird M, et al. Serological Evidence of MERS-CoV Antibodies in Dromedary Camels (Camelus dromedaries) in Laikipia County, Kenya. *PLoS One*. 2015;10(10):e0140125-. doi:10.1371/journal.pone.0140125

51. Ergonul O, Whitehouse CA. Introduction. *Crimean-Congo Hemorrhagic Fever: A Global Perspect*. Published online 2007:3-11. doi:10.1007/978-1-4020-6106-6_1

52. Sorvillo TE, Rodriguez SE, Hudson P, et al. Towards a Sustainable One Health Approach to Crimean-Congo Hemorrhagic Fever Prevention: Focus Areas and Gaps in Knowledge. *Trop Med Infect Dis*. 2020;5(3). doi:10.3390/tropicalmed5030113

53. Mirazimi A, Burt F, Papa A. Crimean-Congo Hemorrhagic Fever Virus and Nairoviruses of Medical Importance (Nairoviridae). In: *Encyclopedia of Virology: Volume 1-5, Fourth Edition*. Vol 1-5. Elsevier; 2020:208-217. doi:10.1016/B978-0-12-814515-9.00036-9

54. Rocque RJ, Beaudoin C, Ndjaboue R, et al. Health effects of climate change: an overview of systematic reviews. *BMJ Open*. 2021;11(6):e046333-. doi:10.1136/bmjopen-2020-046333

55. Cunningham AA, Daszak P, Wood JLN. One Health, emerging infectious diseases and wildlife: two decades of progress? *Philos Trans R Soc Lond B Biol Sci*. 2017;372(1725). doi:10.1098/rstb.2016.0167

56. Lule SA, Gibb R, Kizito D, et al. Widespread exposure to Crimean-Congo haemorrhagic fever in Uganda might be driven by transmission from Rhipicephalus ticks: Evidence from cross-sectional and modelling studies. *J Infect*. 2022;85(6):683-692. doi:10.1016/j.jinf.2022.09.016

57. Othieno J, Njagi O, Masika S, et al. Knowledge, attitudes, and practices on camel respiratory diseases and conditions in Garissa and Isiolo, Kenya. *Front Vet Sci*. 2022;9. doi:10.3389/fvets.2022.1022146

58. Chiuya T, Masiga DK, Falzon LC, Bastos ADS, Fèvre EM, Villinger J. Tick-borne pathogens, including Crimean-Congo haemorrhagic fever virus, at livestock markets and slaughterhouses in western Kenya. *Transbound Emerg Dis*. 2021;68(4):2429-2445. doi:10.1111/tbed.13911

59. Roess A, Carruth L, Mann M, et al. Livestock movement and emerging zoonotic disease outbreaks: applying ecological, network, and sociocultural theories to assess the risk of Middle East respiratory syndrome from camel trade in Ethiopia and Egypt. *Lancet Global Health*. 2015;3:26.

60. Tigoi C, Lwande O, Orindi B, Irura Z, Ongus J, Sang R. Seroepidemiology of selected arboviruses in febrile patients visiting selected health facilities in the lake/river basin areas of Lake Baringo, Lake Naivasha, and Tana River, Kenya. *Vector Borne Zoonotic Dis*. 2015;15(2):124-132. doi:10.1089/vbz.2014.1686

61. Corman VM, Jores J, Meyer B, et al. Antibodies against MERS coronavirus in dromedary camels, Kenya, 1992-2013. *Emerg Infect Dis*. 2014;20(8):1319-1322. doi:10.3201/eid2008.140596

62. Faye B. TADs in the dromedary. In: *Transboundary Animal Diseases in Sahelian Africa and Connected Regions*. Springer International Publishing; 2019:91-103. doi:10.1007/978-3-030-25385-1_6

63. Gikonyo S, Kimani T, Matere J, et al. Mapping Potential Amplification and Transmission Hotspots for MERS-CoV, Kenya. *Ecohealth*. 2018;15(2):372-387. doi:10.1007/s10393-018-1317-6

64. Memish Z. Mers-CoV: From camels to humans. *International Journal of Infectious Diseases*. 2016;45((Memish Z.) Ministry of Health, Riyadh, Saudi Arabia):7-8. doi:10.1016/j.ijid.2016.02.047

65. Bower H, El Karsany M, Alzain M, et al. Detection of Crimean-Congo Haemorrhagic Fever cases in a severe undifferentiated febrile illness outbreak in the Federal Republic of Sudan: A retrospective epidemiological and diagnostic cohort study. *PLoS Negl Trop Dis*. 2019;13(7). doi:10.1371/journal.pntd.0007571

66. Aradaib IE, Erickson BR, Mustafa ME, et al. Nosocomial outbreak of Crimean-Congo hemorrhagic fever, Sudan. *Emerg Infect Dis*. 2010;16(5):837-839. doi:10.3201/eid1605.091815

67. Younan M, Bornstein S, Gluecks I V. MERS and the dromedary camel trade between Africa and the Middle East. *Trop Anim Health Prod*. 2016;48(6):1277-1282. doi:10.1007/s11250-016-1089-3

68. Horton KC, Fahmy NT, Watany N, et al. Crimean Congo Hemorrhagic Fever Virus and Alkhurma (Alkhumra) Virus in Ticks in Djibouti. *Vector Borne Zoonotic Dis*. 2016;16(10):680-682. doi:10.1089/vbz.2016.1951

69. Rahden P, Adam A, Mika A, Jassoy C. Elevated Human Crimean-Congo Hemorrhagic Fever Virus Seroprevalence in Khashm el Girba, Eastern Sudan. *Am J Trop Med Hyg*. 2019;100(6):1549-1551. doi:10.4269/ajtmh.18-0977

70. Ayebare D, Menya M, Mulyowa A, Muhwezi A, Tweyongyere R, Atim SA. Knowledge, attitudes, and practices of Crimean Congo hemorrhagic fever among livestock value chain actors in Kagadi district, Uganda. *PLoS Negl Trop Dis*. 2023;17(2):e0011107-. doi:10.1371/journal.pntd.0011107

71. Adam IA, Mahmoud MA, Aradaib IE. A seroepidemiological survey of Crimean Congo hemorrhagic fever among cattle in North Kordufan State, Sudan. *Virol J*. 2013;10:178. doi:10.1186/1743-422X-10-178

72. Sang RC, Dunster LM. The growing threat of arbovirus transmission and outbreaks in Kenya: a review. *East Afr Med J*. 2001;78(12):655-661. doi:10.4314/eamj.v78i12.8936

73. Hughes EC, Anderson NE. Zoonotic Pathogens of Dromedary Camels in Kenya: A Systematised Review. *Vet Sci*. 2020;7(3). doi:10.3390/vetsci7030103

74. Sitawa R, Folorunso F, Obonyo M, et al. Risk factors for serological evidence of MERS-CoV in camels, Kenya, 2016-2017. *Prev Vet Med*. 2020;185:105197. doi:10.1016/j.prevetmed.2020.105197

75. Atim SA, Niebel M, Ashraf S, et al. Prevalence of Crimean-Congo haemorrhagic fever in livestock following a confirmed human case in Lyantonde district, Uganda. *Parasit Vectors*. 2023;16(1):7. doi:10.1186/s13071-022-05588-x

76. Omrani AS, Al-Tawfiq JA, Memish ZA. Middle East respiratory syndrome coronavirus (MERS-CoV): animal to human interaction. *Pathog Glob Health*. 2015;109(8):354-362. doi:10.1080/20477724.2015.1122852

77. Mohd HA, Al-Tawfiq JA, Memish ZA. Middle East Respiratory Syndrome Coronavirus (MERS-CoV) origin and animal reservoir. *Virol J*. 2016;13:87. doi:10.1186/s12985-016-0544-0

78. Zanella JRC. Environmental Viruses in Livestock Production. In: *Environmental and Food Virology: Impacts and Challenges in One Health Approach*. CRC Press; 2023:44-58. doi:10.1201/9781003263494-3

79. Srivastava D, Kutikuppala LVS, Shanker P, et al. The neglected continuously emerging Marburg virus disease in Africa: A global public health threat. *Health Sci Rep*. 2023;6(11). doi:10.1002/hsr2.1661

80. Towner JS, Nyakarahuka L, Atimnedi P. *Bat-Borne Pathogens and Public Health in Rural African Artisanal Gold Mines*. Vol 26.; 2024.

81. Hunter N, Rathish B. Marburg Fever. Published online 2023.

82. Mitu RA, Islam MR. The Current Pathogenicity and Potential Risk Evaluation of Marburg Virus to Cause Mysterious “Disease X”—An Update on Recent Evidences. *Environ Health Insights*. 2024;18. doi:10.1177/11786302241235809

83. Mane Manohar MP, Lee VJ, Chinedum Odunukwe EU, Singh PK, Mpofu BS, Oxley, MD C. Advancements in Marburg (MARV) Virus Vaccine Research With Its Recent Reemergence in Equatorial Guinea and Tanzania: A Scoping Review. *Cureus*. Published online July 17, 2023. doi:10.7759/cureus.42014

84. Sibomana O, Kubwimana E. First-ever Marburg virus disease outbreak in Equatorial Guinea and Tanzania: An imminent crisis in West and East Africa. *Immun Inflamm Dis*. 2023;11(8). doi:10.1002/iid3.980

85. Sinnott,, John; Kim, Kami; Somboonwit, Charurut; Cosnett, Connor; Segal, David; Shapshak P. Emergent Risk Group-4 (RG-4) Filoviruses: A paradox in progress. *Biomedical Informatics*. 2023;19(8).

86. Ahmed I, Salsabil L, Hossain MJ, Shahriar M, Bhuiyan MA, Islam MR. The recent outbreaks of Marburg virus disease in African countries are indicating potential threat to the global public health: Future prediction from historical data. *Health Sci Rep*. 2023;6(7):e1395-. doi:10.1002/hsr2.1395

87. Rugarabamu SE. Viral haemorrhagic fevers in Tanzania: Seroprevalence and associated risk factors. *Tanzan J Health Res*. 2022;23((Rugarabamu S.E., sima.rugarabamu@sacids.org) Sokoine University of Agriculture, Tanzania):191. doi:10.4314/thrb.v23i1.1S

88. Rugarabamu S, Mwanyika GO, Rumisha SF, et al. Seroprevalence and associated risk factors of selected zoonotic viral hemorrhagic fevers in Tanzania. *Int J Infect Dis*. 2021;109:174-181. doi:10.1016/j.ijid.2021.07.006

89. Nyakarahuka L, Schafer IJ, Balinandi S, et al. A retrospective cohort investigation of seroprevalence of Marburg virus and ebolaviruses in two different ecological zones in Uganda. *BMC Infect Dis*. 2020;20(1):461. doi:10.1186/s12879-020-05187-0

90. Nyakarahuka L, Tumusiime A, Balinandi S, et al. A retrospective cohort study of seroprevalence of Ebola and Marburg viruses in humans from two different ecological zones in Uganda. *American Journal of Tropical Medicine and Hygiene*. 2017;95(5):437.

91. Farnon EC, Adjemian JA, Kansiime E, et al. Filovirus serosurvey following an outbreak of marburg hemorrhagic fever - Ibanda and Kamwenge Districts, Uganda, 2007. *American Journal of Tropical Medicine and Hygiene*. 2009;81(5):209. https://www.embase.com/search/results?subaction=viewrecord&id=L70337202&from=export

92. Adjemian J, Farnon EC, Tschioko F, et al. Outbreak of Marburg hemorrhagic fever among miners in Kamwenge and Ibanda Districts, Uganda, 2007. *J Infect Dis*. 2011;204 Suppl 3(Suppl 3):S796-9. doi:10.1093/infdis/jir312

93. Mangu CD, Manyama CK, Sudi L, et al. Emerging viral infectious disease threat: Why Tanzania is not in a safe zone. *Tanzan J Health Res*. 2016;18(3). doi:10.4314/thrb.v18i3.8

94. Nantima N, Ilukor J, Kaboyo W, et al. The importance of a One Health approach for prioritising zoonotic diseases to focus on capacity-building efforts in Uganda. *REVUE SCIENTIFIQUE ET TECHNIQUE-OFFICE INTERNATIONAL DES EPIZOOTIES*. 2019;38(1):315-325. doi:10.20506/rst.38.1.2963

95. Fhogartaigh CN, Aarons E. Viral haemorrhagic fever. *Clinical Medicine*. 2015;15(1):61-66. doi:10.7861/clinmedicine.15-1-61

96. Changula K, Kajihara M, Mweene AS, Takada A. Ebola and Marburg virus diseases in Africa: Increased risk of outbreaks in previously unaffected areas? *Microbiol Immunol*. 2014;58(9):483-491. doi:10.1111/1348-0421.12181

97. Knust B, Schafer IJ, Wamala J, et al. Multidistrict Outbreak of Marburg Virus Disease - Uganda, 2012. *Journal of Infectious Diseases*. 2015;212((Knust B., bknust@cdc.gov; Schafer I.J.; Dodd K.; Gibbons A.; Campbell S.; Nichol S.T.; Ströher U.; Rollin P.E.) Viral Special Pathogens Branch, Centers for Disease Control and Prevention, MS G-14, 1600 Clifton Rd NE, Atlanta, GA, United States(Lasry E.)):S119-S128. doi:10.1093/infdis/jiv351

98. Mounika G, Sruthi P, Nandini P, Kumar B V, Deepthi BVP. Marburg Virus Disease. *Int J Pharm Sci Rev Res*. 2022;72(2):66-72. doi:10.47583/ijpsrr.2022.v72i02.010

99. Nakazibwe C. Marburg fever outbreak leads scientists to suspected disease reservoir. *Bull World Health Organ*. 2007;85(9):654-656. doi:10.2471/blt.07.020907

100. Towner JS, Amman BR, Sealy TK, et al. Isolation of genetically diverse Marburg viruses from Egyptian fruit bats. *PLoS Pathog*. 2009;5(7):e1000536-. doi:10.1371/journal.ppat.1000536

101. Amman BR, Carroll SA, Reed ZD, et al. Seasonal pulses of Marburg virus circulation in juvenile Rousettus aegyptiacus bats coincide with periods of increased risk of human infection. *PLoS Pathog*. 2012;8(10):e1002877-. doi:10.1371/journal.ppat.1002877

102. Mbonye A, Wamala J, Winyi-Kaboyo, Tugumizemo V, Aceng J, Makumbi I. Repeated outbreaks of viral hemorrhagic fevers in Uganda. *Afr Health Sci*. 2012;12(4):579-583. doi:10.4314/ahs.v12i4.31

103. Amman BR, Jones ME, Sealy TK, et al. Oral shedding of Marburg virus in experimentally infected Egyptian fruit bats (Rousettus aegyptiacus). *J Wildl Dis*. 2015;51(1):113-124. doi:10.7589/2014-08-198

104. Namusisi S, Mahero M, Travis D, Pelican K, Robertson C, Mugisha L. A descriptive study of zoonotic disease risk at the human-wildlife interface in a biodiversity hot spot in South Western Uganda. *PLoS Negl Trop Dis*. 2021;15(1):e0008633-. doi:10.1371/journal.pntd.0008633

105. Idris I. Outbreak of Marburg virus in Tanzania: The need for a major public health response. *Trop Doct*. Published online 2023:494755231169311-. doi:10.1177/00494755231169311

106. Deb N, Roy P, Jaiswal V, Mohanty A, Sah S, Sah R. Marburg Virus Disease in Tanzania: The most recent outbreak. *New Microbes New Infect*. 2023;53:101123. doi:10.1016/j.nmni.2023.101123

107. Nyakarahuka L, Ayebare S, Mosomtai G, et al. Ecological Niche Modeling for Filoviruses: A Risk Map for Ebola and Marburg Virus Disease Outbreaks in Uganda. *PLoS Curr*. 2017;9. doi:10.1371/currents.outbreaks.07992a87522e1f229c7cb023270a2af1

108. Kuzmin I V, Niezgoda M, Franka R, et al. Marburg virus in fruit bat, Kenya. *Emerg Infect Dis*. 2010;16(2):352-354. doi:10.3201/eid1602.091269

109. Willoughby AR, Phelps KL, Olival KJ. A comparative analysis of viral richness and viral sharing in cave-roosting bats. *Diversity (Basel)*. 2017;9(3). doi:10.3390/d9030035

110. Polonsky JA, Wamala JF, de Clerck H, et al. Perspective Piece Emerging Filoviral Disease in Uganda: Proposed Explanations and Research Directions. *AMERICAN JOURNAL OF TROPICAL MEDICINE AND HYGIENE*. 2014;90(5):790-793. doi:10.4269/ajtmh.13-0374

111. Miraglia CM. Marburgviruses: An Update. *Lab Med*. 2019;50(1):16-28. doi:10.1093/labmed/lmy046

112. Bourgarel M, Liegeois F. *Ebola and Other Haemorrhagic Fevers*.; 2019. doi:10.1007/978-3-030-25385-1_1010.1007/978-3-030-25385-1

113. Bulimbe DB, Masunga DS, Paul IK, et al. Marburg virus disease outbreak in Tanzania: current efforts and recommendations - a short communication. *Ann Med Surg (Lond)*. 2023;85(8):4190-4193. doi:10.1097/ms9.0000000000001063

114. Markotter W, Coertse J, L DV, Geldenhuys M, Mortlock M. Bat-borne viruses in Africa: a critical review. *J Zool (1987)*. 2020;311(2):77-98. doi:10.1111/jzo.12769

115. Pigott DM, Golding N, Mylne A, et al. Mapping the zoonotic niche of Marburg virus disease in Africa. *Trans R Soc Trop Med Hyg*. 2015;109(6):366-378. doi:10.1093/trstmh/trv024

116. Asad A, Aamir A, Qureshi NE, et al. Past and current advances in Marburg virus disease: a review. *Infez Med*. 2020;28(3):332-345.

117. Ndjoyi-Mbiguino A, Zoa-Assoumou S, Mourembou G, Ennaji MM. *Ebola and Marburg Virus: A Brief Review*.; 2020. doi:10.1016/B978-0-12-819400-3.00011-910.1016/C2018-0-04146-8

118. Estrada-Peña A, Ostfeld RS, Peterson AT, Poulin R, J de la F. Effects of environmental change on zoonotic disease risk: an ecological primer. *Trends Parasitol*. 2014;30(4):205-214. doi:10.1016/j.pt.2014.02.003

119. Brauburger K, Hume AJ, Mühlberger E, Olejnik J. Forty-five years of marburg virus research. *Viruses*. 2012;4(10):1878-1927. doi:10.3390/v4101878

120. Adesola RO, Warsame AAA, Idris I. Current status of Crimean-Congo hemorrhagic fever outbreaks in Uganda and other African countries. *Health Sci Rep*. 2023;6(7):e1383-. doi:10.1002/hsr2.1383

121. Telford C, Nyakarahuka L, Waller L, Kitron U, Shoemaker T. Spatial prediction of Crimean Congo hemorrhagic fever virus seroprevalence among livestock in Uganda. *One Health*. 2023;17. doi:10.1016/j.onehlt.2023.100576

122. Atim SA, Ashraf S, Belij-Rammerstorfer S, et al. Risk factors for Crimean-Congo Haemorrhagic Fever (CCHF) virus exposure in farming communities in Uganda. *J Infect*. 2022;85(6):693-701. doi:10.1016/j.jinf.2022.09.007

123. Balinandi S, C von B, Tumusiime A, et al. Serological and molecular study of Crimean-Congo Hemorrhagic Fever Virus in cattle from selected districts in Uganda. *J Virol Methods*. 2021;290:114075. doi:10.1016/j.jviromet.2021.114075

124. Jagadesh S, Combe M, Nacher M, Gozlan R. In search for the hotspots of Disease X: A biogeographic approach to mapping the predictive risk of WHO’s blueprint priority diseases. *International Journal of Infectious Diseases*. 2020;101((Jagadesh S.; Combe M.; Gozlan R.) France(Nacher M.) France):220. doi:10.1016/j.ijid.2020.11.013

125. Ogoti BM, Riitho V, Wildemann J, et al. Biphasic MERS-CoV Incidence in Nomadic Dromedaries with Putative Transmission to Humans, Kenya, 2022-2023. *Emerg Infect Dis*. 2024;30(3):581-585. doi:10.3201/eid3003.231488

126. Gardner EG, Kiambi S, Sitawa R, et al. Force of infection of Middle East respiratory syndrome in dromedary camels in Kenya. *Epidemiol Infect*. 2019;147:e275-. doi:10.1017/S0950268819001663

127. Miguel E, Chevalier V, Ayelet G, et al. Risk factors for MERS coronavirus infection in dromedary camels in Burkina Faso, Ethiopia, and Morocco, 2015. *Euro Surveill*. 2017;22(13). doi:10.2807/1560-7917.ES.2017.22.13.30498

128. Zhou Z, Ali A, Walelign E, et al. Genetic diversity and molecular epidemiology of Middle East Respiratory Syndrome Coronavirus in dromedaries in Ethiopia, 2017-2020. *Emerg Microbes Infect*. 2023;12(1):e2164218-. doi:10.1080/22221751.2022.2164218

129. Sang R, Lutomiah J, Koka H, et al. Crimean-Congo hemorrhagic fever virus in Hyalommid ticks, northeastern Kenya. *Emerg Infect Dis*. 2011;17(8):1502-1505. doi:10.3201/eid1708.102064

130. Ngere I, Munyua P, Harcourt J, et al. High MERS-CoV seropositivity associated with camel herd profile, husbandry practices and household socio-demographic characteristics in Northern Kenya. *Epidemiol Infect*. 2020;148:e292-. doi:10.1017/S0950268820002939

131. Wirsiy FS, Nkfusai CN, Bain LE. The SPIN framework to control and prevent the Marburg virus disease outbreak in Equatorial Guinea. *Pan Afr Med J*. 2023;44:110. doi:10.11604/pamj.2023.44.110.39368

132. Megenas JA, Mengistu L, Gezahegne M. Seroprevalence and associated risk factors of Rift Valley fever and Crimean Congo hemorrhagic fever viruses in livestock and their zoonotic potentials: a systematic review. *Veterinaria (Sarajevo)*. 2022;71(1):1-16. doi:10.51607/22331360.2022.71.1.1

133. Kiambi S, Corman VM, Sitawa R, et al. Detection of distinct MERS-Coronavirus strains in dromedary camels from Kenya, 2017. *Emerg Microbes Infect*. 2018;7(1):195. doi:10.1038/s41426-018-0193-z

134. Balinandi S, Patel K, Ojwang J, et al. Investigation of an isolated case of human Crimean-Congo hemorrhagic fever in Central Uganda, 2015. *Int J Infect Dis*. 2018;68:88-93. doi:10.1016/j.ijid.2018.01.013

135. Siya A, Bazeyo W, Tuhebwe D, et al. Lowland grazing and Marburg virus disease (MVD) outbreak in Kween district, Eastern Uganda. *BMC Public Health*. 2019;19(1):136. doi:10.1186/s12889-019-6477-y

136. Rodarte KA, Fair JM, Bett BK, Kerfua SD, Fasina FO, Bartlow AW. A scoping review of zoonotic parasites and pathogens associated with abattoirs in Eastern Africa and recommendations for abattoirs as disease surveillance sites. *Front Public Health*. 2023;11:1194964. doi:10.3389/fpubh.2023.1194964

137. Chretien JP, Anyamba A, Small J, Tucker CJ, Britch SC, Linthicum KJ. Environmental biosurveillance for epidemic prediction: Experience with rift valley fever. In: *Lect. Notes Comput. Sci.* Vol 5354 LNBI. ; 2008:169-174. doi:10.1007/978-3-540-89746-0_17

138. Kanankege KST, Phelps NBD, Vesterinen HM, et al. Lessons Learned From the Stakeholder Engagement in Research: Application of Spatial Analytical Tools in One Health Problems. *Front Vet Sci*. 2020;7:254. doi:10.3389/fvets.2020.00254

139. Pascoe EL, Pareeth S, Rocchini D, Marcantonio M. A lack of “environmental earth data” at the microhabitat scale impacts efforts to control invasive arthropods that vector pathogens. *Data (Basel)*. 2019;4(4). doi:10.3390/data4040133

140. Habib S, Policelli F, Irwin D, Korme T, Adler B, Hong Y. Application of satellite observations to manage natural disasters in the Lake Victoria Basin. In: *Dig Int Geosci Remote Sens Symp (IGARSS)*. Vol 4. ; 2009:IV21-IV24. doi:10.1109/IGARSS.2009.5417607

141. Zhang AR, Li XL, Wang T, et al. Ecology of Middle East respiratory syndrome coronavirus, 2012-2020: A machine learning modelling analysis. *Transbound Emerg Dis*. 2022;69(5):e2122-e2131. doi:10.1111/tbed.14548

142. Lash RR, Brunsell NA, Peterson AT. Spatiotemporal environmental triggers of Ebola and Marburg virus transmission. *Geocarto Int*. 2008;23(6):451-466. doi:10.1080/10106040802121010

143. Peterson AT, Bauer JT, Mills JN. Ecologic and Geographic Distribution of Filovirus Disease. *Emerg Infect Dis*. 2004;10(1):40. doi:10.3201/EID1001.030125

144. Marin-Ferrer K, Vernaccini M, Messina L. Incorporating epidemics risk in the INFORM Hazard-dependent Global Risk Index, EUR 29603 EN, Publications Office of the European Union. In: ; 2018:114652. doi:10.2760/990429

145. Pigott DM, Deshpande A, Letourneau I, et al. Local, national, and regional viral haemorrhagic fever pandemic potential in Africa: a multistage analysis. *Lancet*. 2017;390(10113):2662-2672. doi:10.1016/S0140-6736(17)32092-5

146. Wang H, Zeng J, Gao X, Wang H, Xiao J. SpatMCDA: An R package for assessing areas at risk of infectious diseases based on spatial multi-criteria decision analysis. *Methods Ecol Evol*. Published online 2024. doi:10.1111/2041-210X.14364

147. World Health Organization. *Health Emergency and Disaster Risk Management Framework*.; 2019.

148. UN Office for Disaster Risk Reduction. *Technical Guidance on Application of Climate Information for Comprehensive Risk Management*.; 2023. https://www.undrr.org/contact-us.

149. Onyango EA, Sahin O, Awiti A, Chu C, Mackey B. An integrated risk and vulnerability assessment framework for climate change and malaria transmission in East Africa. *Malar J*. 2016;15(1):551. doi:10.1186/s12936-016-1600-3

150. Abutarbush SM, Hamdallah A, Hawawsheh M, et al. Implementation of one health approach in Jordan: Joint risk assessment of rabies and avian influenza utilizing the tripartite operational tool. *One Health*. 2022;15:100453. doi:10.1016/j.onehlt.2022.100453

151. S de la R, Errecaborde KMM, Belot G, et al. One health systems strengthening in countries: Tripartite tools and approaches at the human-animal-environment interface. *BMJ Glob Health*. 2023;8(1). doi:10.1136/bmjgh-2022-011236

152. Grange ZL, Goldstein T, Johnson CK, et al. Ranking the risk of animal-to-human spillover for newly discovered viruses. *Proc Natl Acad Sci U S A*. 2021;118(15):e2002324118. doi:10.1073/PNAS.2002324118/SUPPL_FILE/PNAS.2002324118.SAPP.PDF

153. Peterson AT, Papeş M, Carroll DS, Leirs H, Johnson KM. Mammal taxa constituting potential coevolved reservoirs of filoviruses. *J Mammal*. 2007;88(6):1544-1554. doi:10.1644/06-MAMM-A-280R1.1

154. Munyua P, Bitek A, Osoro E, et al. Prioritization of zoonotic diseases in Kenya, 2015. *PLoS One*. 2016;11(8). doi:10.1371/journal.pone.0161576

155. Baldassi F, Cenciarelli O, Malizia A, Gaudio P. First Prototype of the Infectious Diseases Seeker (IDS) Software for Prompt Identification of Infectious Diseases. *J Epidemiol Glob Health*. 2020;10(4):367-377. doi:10.2991/jegh.k.200714.001

156. Baldo A, Welby S, Thi CDD, Leunda A, Herman P, Breyer D. Risk Classification of Zoonotic Microorganisms: Development of a One Health Approach Tool Applied to HPAI A H5N1 Viruses. *Appl Biosafety*. 2018;23(4):199-210. doi:10.1177/1535676018799094

157. Hwang J, Lee K, Walsh D, Kim SW, Sleeman JM, Lee H. Semi-quantitative assessment of disease risks at the human, livestock, wildlife interface for the Republic of Korea using a nationwide survey of experts: A model for other countries. *Transbound Emerg Dis*. 2018;65(1):e155-e164. doi:10.1111/tbed.12705

158. Africa Centers for Disease Control and Prevention. *Risk Ranking and Prioritization of Epidemic-Prone Diseases*.; 2023.

159. Singh M, Periasamy A, Goyal P, Goswami S, Yadav A. Environmental risk assessment for Zika, Nipah virus and Scrub typhus disease in a district of north India: First step towards one health. *J Zoonotic Dis*. 2022;6(1):25-32. doi:10.22034/JZD.2022.14408

160. Wang X, Wu F, Zhao X, et al. Enlightenment from the COVID-19 Pandemic: The Roles of Environmental Factors in Future Public Health Emergency Response. *Engineering*. 2022;8:108-115. doi:10.1016/j.eng.2020.12.019

161. Gwenzi W, Skirmuntt EC, Musvuugwa T, Teta C, Halabowski D, Rzymski P. Grappling with (re)-emerging infectious zoonoses: Risk assessment, mitigation framework, and future directions. *Int J Disaster Risk Reduct*. 2022;82. doi:10.1016/j.ijdrr.2022.103350

162. Su Y, Gao R, Huang F, et al. Occurrence, transmission and risks assessment of pathogens in aquatic environments accessible to humans. *J Environ Manage*. 2024;354. doi:10.1016/j.jenvman.2024.120331

163. Terzi S, Torresan S, Schneiderbauer S, Critto A, Zebisch M, Marcomini A. Multi-risk assessment in mountain regions: A review of modelling approaches for climate change adaptation. *J Environ Manage*. 2019;232:759-771. doi:10.1016/j.jenvman.2018.11.100

164. Muthamizharasan M, Ponnusamy R. A Hybrid CNN and GRU-based Spatial-temporal Marburg Virus Disease Hotspot Association Mining for Health Management in Kenya. In: *Int. Conf. Data Sci., Agents Artif. Intel., ICDSAAI*. Institute of Electrical and Electronics Engineers Inc.; 2022. doi:10.1109/ICDSAAI55433.2022.10028852

165. Deka MA. Crimean-Congo Hemorrhagic Fever Geographic and Environmental Risk Assessment in the Balkan and Anatolian Peninsulas. *Pap Appl Geogr*. 2018;4(1):46-71. doi:10.1080/23754931.2017.1378122

166. Messina JP. The global distribution of Crimean-Congo hemorrhagic fever. *American Journal of Tropical Medicine and Hygiene*. 2013;89(5):18-19. https://www.embase.com/search/results?subaction=viewrecord&id=L71311892&from=export

167. Escobar LE, Peterson AT, Papeş M, et al. Ecological approaches in veterinary epidemiology: mapping the risk of bat-borne rabies using vegetation indices and night-time light satellite imagery. *Vet Res*. 2015;46(1):92. doi:10.1186/s13567-015-0235-7

168. Temur AI, Kuhn JH, Pecor DB, Apanaskevich DA, Keshtkar-Jahromi M. Epidemiology of Crimean-Congo Hemorrhagic Fever (CCHF) in Africa-Underestimated for Decades. *Am J Trop Med Hyg*. 2021;104(6):1978-1990. doi:10.4269/ajtmh.20-1413

169. Wille M, Geoghegan JL, Holmes EC. How accurately can we assess zoonotic risk? *PLoS Biol*. 2021;19(4):e3001135-. doi:10.1371/journal.pbio.3001135

170. Wilkinson DA, Marshall JC, French NP, Hayman DTS. Habitat fragmentation, biodiversity loss and the risk of novel infectious disease emergence. *J R Soc Interface*. 2018;15(149). doi:10.1098/rsif.2018.0403

171. Roberts M, Dobson A, Restif O, Wells K. Challenges in modelling the dynamics of infectious diseases at the wildlife–human interface. *Epidemics*. 2021;37((Roberts M., m.g.roberts@massey.ac.nz) School of Natural & Computational Sciences, New Zealand Institute for Advanced Study and the Infectious Disease Research Centre, Massey University, Private Bag 102 904, North Shore Mail Centre, Auckland, New Zealand(). doi:10.1016/j.epidem.2021.100523

172. Purse B V, Golding N. Tracking the distribution and impacts of diseases with biological records and distribution modelling. *Biol J Linn Soc*. 2015;115(3):664-677. doi:10.1111/bij.12567

173. Thrush MA, Murray AG, Brun E, Wallace S, Peeler EJ. The application of risk and disease modelling to emerging freshwater diseases in wild aquatic animals. *Freshw Biol*. 2011;56(4):658-675. doi:10.1111/j.1365-2427.2010.02549.x

174. Ogden NH, Robbin Lindsay L, Drebot MA. Zoonoses. In: *Climate Change and Animal Health*. CRC Press; 2022:141-155. doi:10.1201/9781003149774-7

175. Escobar LE, Craft ME. Advances and Limitations of Disease Biogeography Using Ecological Niche Modeling. *Front Microbiol*. 2016;7:1174. doi:10.3389/fmicb.2016.01174

176. Corley CD, Pullum LL, Hartley DM, et al. Disease prediction models and operational readiness. *PLoS One*. 2014;9(3):e91989-. doi:10.1371/journal.pone.0091989

177. Ahmed A, Ahmed A. GIS and Remote Sensing for Malaria Risk Mapping, Ethiopia. *The International Archives of the Photogrammetry, Remote Sensing and Spatial Information Sciences*. 2014;XL-8(8):155-161. doi:10.5194/ISPRSARCHIVES-XL-8-155-2014

178. Wimberly MM, Bayabil E, Beyene B, et al. EPIDEMIA-An EcoHealth informatics system for integrated forecasting of malaria epidemics. *Malar J*. 2014;13((Beyene B.)(Wimberly M.M.; Henebry G.; Liu Y.; Merkord C.L.) South Dakota State University, Brookings, SD, United States(Bayabil E.; Lemma A.M.; Mihretie A.) Health, Development, and Anti-Malaria Association, Addis Ababa, Ethiopia(Bishaw M.) Gamby College):S38-. https://www.embase.com/search/results?subaction=viewrecord&id=L71644571&from=export

179. Izzah LN, Majid Z, Ariff MAM, Fook CK. Geospatial analysis of urban land use pattern analysis for hemorrhagic fever risk ’ a review. In: *Int. Arch. Photogramm., Remote Sens. Spat. Inf. Sci. - ISPRS Arch.* Vol 42. International Society for Photogrammetry and Remote Sensing; 2016:37-53. doi:10.5194/isprs-archives-XLII-4-W1-37-2016

180. Attaway DF, Jacobsen KH, Falconer A, Manca G, Waters NM. Risk analysis for dengue suitability in Africa using the ArcGIS predictive analysis tools (PA tools). *Acta Trop*. 2016;158:248-257. doi:10.1016/j.actatropica.2016.02.018

181. Hemming D, Macneill K. Use of meteorological data in biosecurity. *Emerg Top Life Sci*. 2020;4(5):497-511. doi:10.1042/ETLS20200078

182. Muylaert RL, Wilkinson DA, Kingston T, et al. Using drivers and transmission pathways to identify SARS-like coronavirus spillover risk hotspots. *bioRxiv*. 2022;((Muylaert R.L., R.deLaraMuylaert@massey.ac.nz; Hayman D.T.S.) Massey University, Palmerston North, New Zealand). doi:10.1101/2022.12.08.518776

183. Zumla A, Dar O, Kock R, et al. Taking forward a “One Health” approach for turning the tide against the Middle East respiratory syndrome coronavirus and other zoonotic pathogens with epidemic potential. *Int J Infect Dis*. 2016;47:5-9. doi:10.1016/j.ijid.2016.06.012

184. Johnson MS, Korcz M, Von Stackelberg K, Hope BK. Spatial analytical techniques for risk based decision support systems. In: *Decis. Support Syst. for Risk-Based Mgmt. of Contaminated Sites*. Springer US; 2009:75-93. doi:10.1007/978-0-387-09722-0_4

185. McIntyre KM, Vogler B, Chantziaras I, et al. *Assessing the Ecological Dimension of One Health*.; 2018. doi:10.3920/978-90-8686-875-9

186. Nyakarahuka L, Knust B, Schafer I, Nzietchueng S, Wamala J, Shoemaker T. Using network analysis technique to describe the spread of Marburg hemorrhagic fever outbreak in Uganda, 2012. *International Journal of Infectious Diseases*. 2014;21((Nyakarahuka L.) Makerere University/Uganda Virus Research Institute, Kampala, Uganda(Knust B.; Schafer I.) CDC, Atlanta, United States(Nzietchueng S.) University of Minnesota, Minneapolis, United States(Wamala J.) Ministry of Health, Kampala, Uganda(Shoe):2. doi:10.1016/j.ijid.2014.03.410

187. Ramadan N, Shaib H. Middle East respiratory syndrome coronavirus (MERS-CoV): A review. *Germs*. 2019;9(1):35-42. doi:10.18683/germs.2019.1155

188. Britch SC, Binepal YS, Ruder MG, et al. Rift Valley fever risk map model and seroprevalence in selected wild ungulates and camels from Kenya. *PLoS One*. 2013;8(6):e66626-. doi:10.1371/journal.pone.0066626

189. Yasobant S, Lekha KS, Saxena D. Risk Assessment Tools from the One Health Perspective: A Narrative Review. *Risk Manag Healthc Policy*. 2024;17:955-972. doi:10.2147/RMHP.S436385

190. Dente MG, Riccardo F, Milano A, et al. A One Health-based Conceptual Framework for comprehensive and coordinated prevention and preparedness to health threats. *International Journal of Infectious Diseases*. 2022;116((Dente M.G.) Istituto Superiore di Sanità, National Center for Global Health, Rome, Italy(Riccardo F.; Villa L.; Monaco M.) Istituto Superiore di Sanità, Infectious diseases Dept., Rome, Italy(Milano A.; Robbiati C.; Declich S.) Istituto Superiore di Sani):S108-S109. doi:10.1016/j.ijid.2021.12.256

191. Dente MG, Riccardo F, W VB, et al. Enhancing Preparedness for Arbovirus Infections with a One Health Approach: The Development and Implementation of Multisectoral Risk Assessment Exercises. *Biomed Res Int*. 2020;2020:4832360. doi:10.1155/2020/4832360

192. Dewar R, Gavin C, McCarthy C, Taylor RA, Cook C, Simons RRL. A user-friendly decision support tool to assist one-health risk assessors. *One Health*. 2021;13((Dewar R., Robert.dewar@apha.gov.uk; Gavin C.; McCarthy C.; Taylor R.A.; Cook C.; Simons R.R.L.) Animal and Plant Health Agency, Woodham Lane, Addlestone, United Kingdom). doi:10.1016/j.onehlt.2021.100266

193. Joint Risk Assessment Operational Tool (JRA OT). Accessed July 30, 2022. https://www.who.int/initiatives/tripartite-zoonosis-guide/joint-risk-assessment-operational-tool

194. United Nations Office for Disaster Risk Reduction (UNDRR). Projecting Effects of Climate Change in the Framework of the INFORM Risk Index. Published online 2022.

195. Pigott DM, Deshpande A, Letourneau I, et al. Local, national, and regional viral haemorrhagic fever pandemic potential in Africa: a multistage analysis. *The Lancet*. 2017;390(10113):2662-2672. doi:10.1016/S0140-6736(17)32092-5

196. Grange ZL, Goldstein T, Johnson CK, et al. Ranking the risk of animal-to-human spillover for newly discovered viruses. *Proc Natl Acad Sci U S A*. 2021;118(15). doi:10.1073/pnas.2002324118

197. World Health Organization. Health Emergency and Disaster Risk Management Framework. Published online 2019.

198. Mazet JA, Clifford DL, Coppolillo PB, Deolalikar AB, Erickson JD, Kazwala RR. A “one health” approach to address emerging zoonoses: the HALI project in Tanzania. *PLoS Med*. 2009;6(12):e1000190-. doi:10.1371/journal.pmed.1000190

199. Zinsstag J, Kaiser-Grolimund A, Heitz-Tokpa K, et al. Advancing One human-animal-environment Health for global health security: what does the evidence say? *Lancet*. 2023;401(10376):591-604. doi:10.1016/S0140-6736(22)01595-1

200. WHO. *WHO Guidance for Climate-Resilient and Environmentally-Sustainable Healthcare Facilities*.; 2020.
